# Split intein-mediated reconstitution of regulatable CAR T-cells

**DOI:** 10.3389/fimmu.2026.1844972

**Published:** 2026-10-02

**Authors:** Vladimir Mulens-Arias, Marco Archinti, Miriam Bastida, Nuria Martínez, Eleonora Pinto, Víctor M. Díaz, Pablo Menéndez

**Affiliations:** 1 Josep Carreras Leukemia Research Institute (IJC), Barcelona, Spain; 2 Red Española de Terapias Avanzadas (TERAV+), Instituto de Salud Carlos III, Madrid, Spain; 3 ZIP Solutions SL., Barcelona, Spain; 4 Department of Clinical Sciences and Translational Medicine, Universityof Rome “Tor Vergata”, Rome, Italy; 5 OneChain Immunotherapeutics SL., Barcelona, Spain; 6 Universitat Internacional de Catalunya, Sant Cugat del Vallès, Spain; 7 Centro de Investigación Biomédica en Red de Cáncer (CIBERONC), Madrid, Spain; 8 Department of Biomedicine, School of Medicine, University of Barcelona, Barcelona, Spain; 9 Institució Catalana de Recerca i Estudis Avançats (ICREA), Barcelona, Spain; 10 Institut de Recerca Hospital Sant Joan de Déu-Pediatric Cancer Center Barcelona (SJD-PCCB), Barcelona, Spain

**Keywords:** CAR T-cells, immunotherapy, modular, protein trans-splicing, split inteins

## Abstract

**Introduction:**

Chimeric antigen receptor (CAR) T-cell therapy has transformed the treatment of B-cell malignancies. However, strategies that improve the modularity and controllability of CAR signaling could broaden its applicability and safety across additional clinical contexts. Here, we describe a regulable CAR T-cell platform based on split intein-mediated protein trans-splicing (PTS) to conditionally reconstitute functional receptors in primary T-cells.

**Methods:**

As a proof of concept, we engineered a split CAR targeting CD1a (NCT05679895), a surface antigen expressed in T-cell acute lymphoblastic leukemia (T-ALL). The system consists of a membrane-anchored C-terminal scaffold containing the IMPDH.1^C^ split intein fused to 4-1BB and CD3ζ signaling domains, and a soluble N-terminal adaptor composed of an anti-CD1a single-chain variable fragment (scFv) fused to the complementary IMPDH.1^N^ split intein.

**Results:**

Upon delivery of the adaptor module, the intein fragments undergo PTS, generating a functional membrane-bound CD1a CAR with splicing efficiencies of 85%. Reconstituted CAR T-cells exhibited antigen-specific cytotoxicity against CD1a^+^ T-ALL cells coupled to high levels of interferon-γ secretion.

**Discussion:**

These findings demonstrate that split intein-mediated CAR reconstitution enables a modular platform for conditional CAR reconstitution and transient control of receptor availability.

## Graphical abstract

**Figure:**
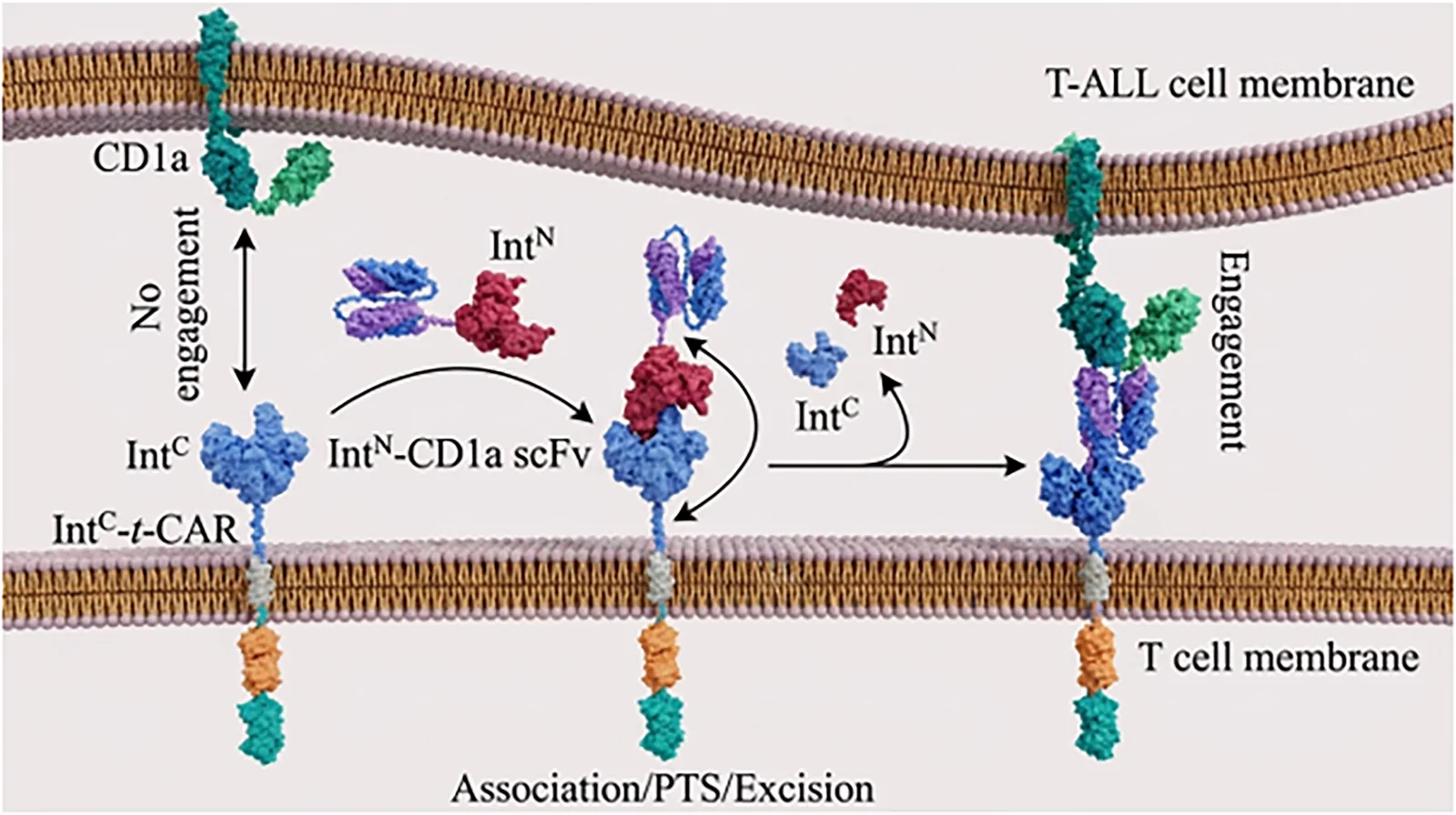

## 1 Introduction

Chimeric antigen receptor (CAR) T-cell therapy has emerged as an effective treatment for several hematological malignancies (1, 2). Following the landmark clinical successes of CD19-targeted CAR T-cells in B-cell malignancies, this approach has achieved unprecedented remission rates in relapsed and refractory leukemias and lymphomas, leading to several approved products (3, 4). These achievements have positioned CAR T-cell therapy as an attractive asset in immunoncology, supporting the exploration of new therapeutic approaches and offering durable remissions in patients with otherwise limited therapeutic options.

Despite these advances, conventional CAR architectures exhibit fixed antigen specificity and signaling configurations once infused into patients. This rigidity constrains real-time modulation of T-cell effector functions and, most importantly, limits the capacity to retarget evolving tumor clones subjected to therapeutic selective pressure. Moreover, current CAR T-cell platforms face persistent challenges, including antigen escape (5), on-target/off-tumor toxicities (6), suboptimal efficacy in solid tumors (7), and manufacturing constraints that hinder scalability and broad clinical adoption (8).

Modular CAR T-cell design strategies aim to overcome these limitations by uncoupling antigen recognition from intracellular signaling (9). Such strategies can permit the dynamic exchange of targeting domains, enable multiplexed antigen recognition, and allow dose-dependent tuning of activity, features that likely reduce toxicity while enhancing durability of anti-tumor responses. By incorporating programmable elements, modular CAR systems can provide a flexible framework capable of addressing tumor heterogeneity and resistance mechanisms. Among these emerging technologies, intein-mediated protein splicing platforms might offer a feasible approach for conditional, post-translational assembly of functional receptors in response to exogenous cues (10–12), thereby enabling controlled CAR activation and improved therapeutic precision.

Inteins are self-excising protein domains that catalyze their own removal from host polypeptides while simultaneously ligating the adjoining sequences, named exteins, through a native peptide bond (13, 14). Engineered split inteins extend this capability by enabling conditional protein reconstitution: when the complementary N- and C-terminal intein fragments encounter the appropriate structural or biochemical environment, they associate and drive protein trans-splicing (PTS), a process that can be further regulated by redox cues (15). In contrast to transcriptional or genome-integrated regulatory systems, PTS offers a unique post-translational mechanism for controlling CAR assembly, allowing direct protein modification without impacting genetic background. This might allow for precise temporal gating of receptor expression and function without altering endogenous gene expression programs, making PTS a feasible strategy for the conditional activation of engineered immune receptors.

Previous research on split intein-based modular CAR systems, known as CARteins, has primarily investigated the intracellular protein trans-splicing-mediated assembly of CARs via co-expression of two distinct sequences (11). Although this approach represents an advance in employing split inteins for CAR system design, it is nonetheless dependent on co-expression of both sequences by the same cell. Furthermore, the feasibility of this system in primary human T-cells remains unstested.

Jiang Y et al. chemically conjugated a C-terminal split intein to an anti-CD19 whole antibody, which served as an external adapter (16). Upon addition to T-cells expressing a membrane-bound N-terminal split intein fused to the T-cell receptor/CD3 complex, the anti-CD19 targeting moiety was efficiently incorporated into the engineered T-cell receptor platform via PTS, resulting in a functional CAR termed ProtinCAR. This approach, however, requires chemical modification of the C-terminal split intein, adding an additional layer of complexity for clinical translation.

Altogether, an important question remains: How do the expression of a truncated receptor in T-cells and the conditional, extracellular assembly of full-length CARs at the protein level through split-intein-mediated protein trans-splicing affect the biology and functional fitness of primary human T-cells?

To address such issues, we employed a bioengineered split intein strategy, specifically the IMPDH.1^C^/IMPDH.1^N^ system, to reconstitute a full-length anti-CD1a CAR, a clinically validated CAR previously developed in our laboratory (NCT05679895) (17, 18), on the surface of human primary T-cells. The platform is built around a truncated membrane-embedded CAR consisting of the CD8α transmembrane region and 4-1BB/CD3ζ signaling modules fused to the C-terminal intein fragment (IMPDH.1^C^). The complementary anti-CD1a single-chain variable fragment (scFv) is produced as a soluble polypeptide fused to the N-terminal intein fragment (IMPDH.1^N^). Upon exogenous delivery, the scFv-IMPDH.1^N^ component undergoes redox-dependent PTS with the membrane-anchored receptor fragment, thereby restoring a fully functional CAR at the cell surface. This configuration might enable precise spatial and temporal control over CAR functionality, allowing “on-demand” receptor assembly dictated by the availability of the N-terminal intein-scFv component. In this proof-of-concept study, we demonstrate efficient reconstitution of functional CARs in human primary T-cells, achieving robust surface expression without perturbing T-cell homeostasis, viability, or phenotypic integrity. Importantly, the reconstituted CAR T-cells exhibit potent, antigen-specific cytotoxicity against CD1a^+^ targets.

## 2 Results

### 2.1 *In silico* modeling confirms the structural feasibility of intein-mediated CAR reconstitution

The rationale for employing a modular CAR platform, understood as the physical separation of the binding domain and the signaling domains, lies in its capacity to confer the possibility of using diverse antigen specificities. Split inteins are an ideal tool for this application due to their highly selective association, which drives the spontaneous folding of a functional three-dimensional (3D) intein structure. This conformational assembly positions catalytic residues in precise proximity, facilitating PTS and the covalent linkage of truncated surface-expressed CARs in T-cells. We designed a second-generation, split-CAR platform in which the extracellular targeting domain (an anti-CD1a scFv) is covalently conjugated to a truncated receptor *via* PTS (**Figure 1A**). An IMPDH.1 split intein pair derived from the intronless inosine-5′-monophosphate dehydrogenase gene of *Methanococcus jannaschii* was evaluated. The truncated CAR construct is designed to lack the antigen-binding domain and instead incorporates the C-terminal fragment of the intein (IMPDH.1^C^-*t*-CAR), thereby enabling reconstitution of a functional, full-length CAR upon interaction with a complementary N-terminal fragment. Lentiviral delivery of the IMPDH.1^C^-*t*-CAR construct encodes a signal peptide, a 6× His-tag to facilitate detection, the C-terminal intein fragment IMPDH.1^C^, CD8α hinge and transmembrane domains, 4-1BB and CD3ζ signaling motifs, and a T2A-linked GFP reporter (**Figure 1B**, **Supplementary Methods**). For the soluble N-terminal counterparts, the N-terminal intein fragment was fused to the CD1a scFv (IMPDH.1^N^-CD1a scFv), previously validated as the binding domain of a functional full-length CAR (18).

**Figure 1:**
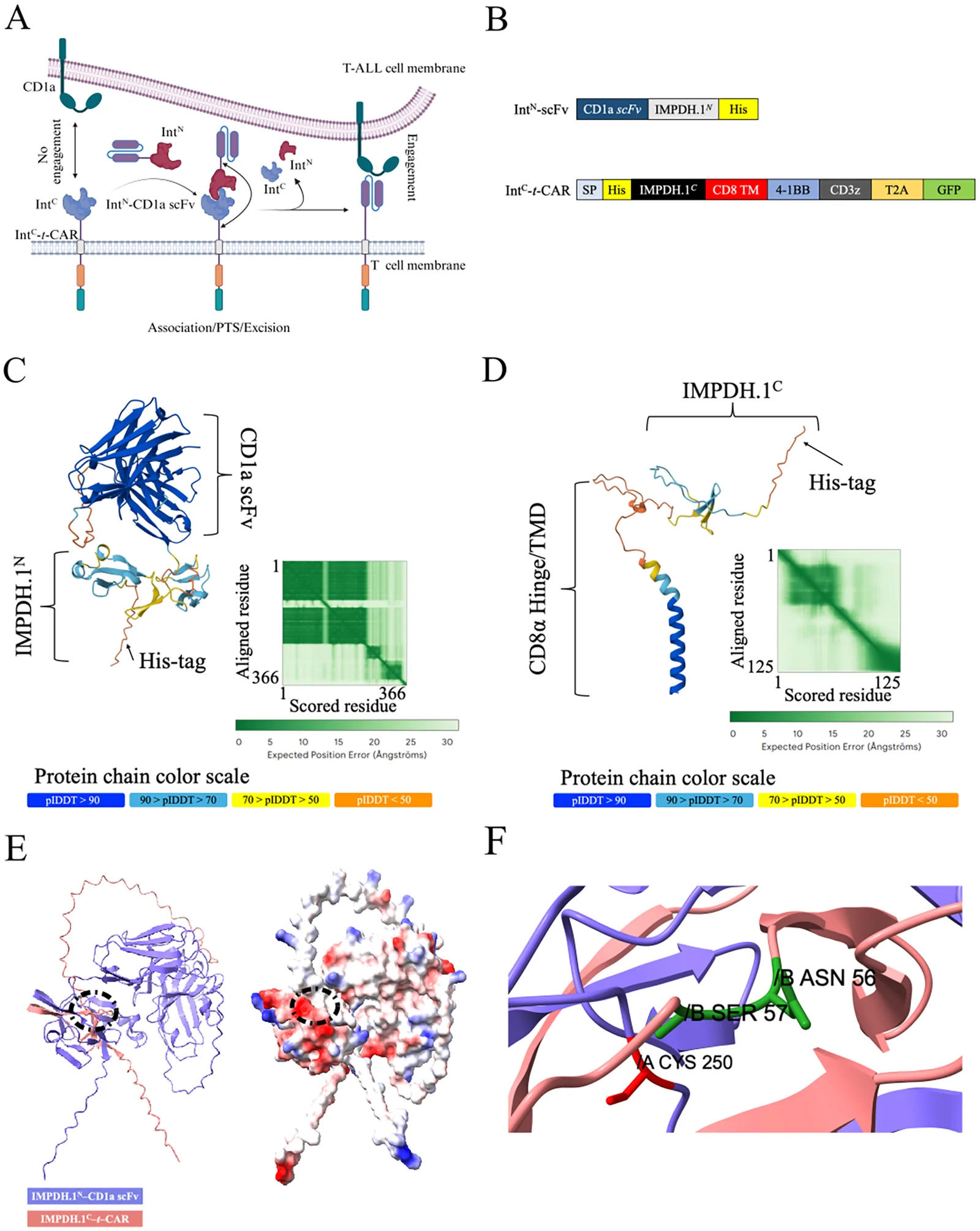
*In silico* analysis of split intein modules in a CD1a-specific regulable CAR. **(A)** Scheme depicting the mechanism of an intein-based split-CAR platform (1). A truncated CAR (Int^C^-*t*-CAR), lacking antigen specificity and harboring an extracellular C-terminal intein fragment, is lentivirally integrated into the T-cell genome and expressed as a transmembrane scaffold (2). A soluble polypeptide consisting of an antigen-recognition scFv domain fused to a complementary N-terminal intein fragment interacts with Int^C^-*t*-CAR-expressing T-cells (3). Interaction between the complementary split inteins drives their association and folding into a β-sheet-rich domain that aligns the catalytic residues required for protein trans-splicing (PTS) (4). The trans-splicing reaction releases the intein fragments and covalently fuses the scFv to the truncated CAR, thereby reconstituting a full-length, functional CAR (5). The reconstituted CAR recognizes its cognate antigen and triggers cytotoxic activity against target leukemia cells. *Scheme created with BioRender™.* **(B)** Overview of the IMPDH.1^N^-CD1a scFv (upper) and IMPDH.1^C^-*t*-CAR (bottom) constructs showing all domains. **(C)** Predicted structure of IMPDH.1^N^-CD1a scFv by AlphaFold v3 and colored by predicted local distance difference test (pLDDT). Model color represents a per-atom confidence estimate; blue: high confidence; orange: low confidence. The model displays two distinct domains: an Ig domain corresponding to the CD1a scFv and a β-sheet-rich domain corresponding to the IMPDH.1^N^ intein. Inset: predicted aligned error (PAE) matrix. Protein structures were predicted with default parameters. **(D)** Predicted structure of IMPDH.1^C^-*t*-CAR (comprising the IMPDH.1^C^, and the CD8α hinge and transmembrane domain) as modeled by AlphaFold v3 and colored by pLDDT as above. The extracellular domain, including the IMPDH.1^C^ intein and CD8α hinge, appears largely disordered, with only a small loop formed by adjacent β-sheets of the IMPDH.1^C^. The CD8α transmembrane domain adopts the expected α-helix conformation. Inset: PAE matrix. Protein structures were predicted with default parameters. **(E)** Predicted interaction between IMPDH.1^N^-CD1a scFv (*magenta*) and IMPDH.1^C^-*t*-CAR (*pink*). The CD1a scFv domain remains structurally conserved, while the split inteins reconstitute into a β-sheet-rich fold characteristic of natural inteins (*left*). Surface charge distribution of the predicted complex is shown (*right*). **(F)** Magnified view of the interaction interface (black dotted circle in E) between IMPDH.1^N^ (*magenta*) and IMPDH.1^C^ (*pink*) domains, highlighting the proximity of catalytic residues required for PTS, namely Cys250 (*red*) and the adjacent Asn56-Ser57 dyad (*green*).

To assess the feasibility of split intein-mediated CAR reconstitution, we performed *in silico* modeling using AlphaFold3 (19). IMPDH.1^N^-CD1a scFv (see sequence in **Supplementary Information**) was predicted to adopt a bifunctional structure, comprising an Ig-like scFv domain and a β-sheet-rich domain characteristic of the native intein fold (**Figure 1C**). The IMPDH.1^C^-*t*-CAR simulation (submitted sequence underlined in **Supplementary Information**) revealed an intact CD8α transmembrane domain, a disordered extracellular CD8α hinge, and the short C-terminal intein fragment retaining two adjacent β-sheets reminiscent of the native fold (**Figure 1D**). Notably, modeling of the split intein interaction predicted preservation of the scFv domain and reconstitution of the canonical β-sheet-rich Hedgehog/Intein (HINT) fold (14) (**Figure 1E**), precisely orienting catalytic residues critical for PTS (**Figure 1F**). AF3 modeling of the split-intein complex showed moderate overall interface confidence (top-ranked model ipTM = 0.49; ranking score = 0.68; **Supplementary Table S1**). Nevertheless, the predicted interaction interface was highly conserved across the five generated models, with mean interface Jaccard indices of 0.86 and 0.90 for the respective interacting chains and a mean residue-pair contact-map Jaccard of 0.79. Moreover, predicted inter-chain contacts displayed relatively low PAE values across models (~3.9–4.6 Å), indicating consistent relative positioning of the interacting residues. Together, these analyses revealed substantial cross-model convergence of the predicted interaction interface despite the moderate global AF3 interface-confidence score.

The interface between the two intein fragments inferred from AF3 displayed a predominantly negatively charged surface (**Figure 1E**, right), facilitating the spatial proximity of key catalytic residues required for autocleavage: Cys250 (IMPDH.1^N^ Cys1) from the Int^N^-CD1a scFv, and the sequential residues His55 (IMPDH.1^C^ His39), Asn56 (IMPDH.1^C^ Asn40), and Ser57 (IMPDH.1^C^ Ser +1) from the IMPDH.1^C^-*t*-CAR (**Figure 1F**). Specifically, Cys250 from the IMPDH.1^N^-CD1a scFv serves as the nucleophile initiating the splicing reaction, promoting the N-S acyl shift to form a highly reactive thioester bond, while Ser57 from IMPDH.1^C^-*t*-CAR acts as the canonical Ser +1 catalytic residue that accepts the CD1a scFv from Cys250. Asn56 from the IMPDH.1^C^-*t*-CAR facilitates the final cyclization step, forming a succinimide intermediate that drives intein excision and ligation of the flanking protein fragments. This arrangement is consistent with a mechanism supporting autocatalytic PTS. Thus, the constructs comprising both pairs of IMPDH.1 split inteins are predicted to efficiently collapse into the native 3D intein conformation and were predicted to reconstitute the full-length CD1a CAR, as supported by *in silico* modeling of the 3D conformations of the individual fragments and their sequence-specific associations.

### 2.2 IMPDH.1^N^-CD1a scFv is efficiently produced, specifically recognizes CD1a and engages IMPDH.1^C^-*t*-CAR in a dose-dependent manner

We then sought to demonstrate *in vitro* the IMPDH.1 split intein-mediated PTS. We next expressed the IMPDH.1^N^-CD1a scFv in TurboCHO cells, achieving yields of 9.5 mg/L of culture. IMAC-purified IMPDH.1^N^-CD1a scFv (50µg/100µl) specifically recognized the CD1a-expressing MOLT-4 cells (**Figure 2A**; **Supplementary Figure S1**). PTS was evaluated using a model IMPDH.1^C^ partner designed to transfer an irrelevant variable heavy-chain (VHH) domain to CD1a scFv upon successful splicing. Specifically, His-tagged IMPDH.1^N^-CD1a scFv was incubated with the small ubiquitin-like modifier (SUMO)-His-IMPDH.1^C^-VHH, enabling the generation of the trans-splicing product (TSP) CD1a scFv-VHH, along with His-tagged IMPDH.1^N^ and SUMO-His-IMPDH.1^C^ by-products. To unambiguously confirm the formation of the TSP, the reaction mixture was applied to a Ni-resin column. Because the TSP lacks a His-tag, it is the only reaction component not expected to bind the resin (**Figure 2B**). The recovery of the untagged CD1a scFv-VHH conjugate in the flow-through demonstrates that the IMPDH.1^N^-CD1a scFv is biochemically reactive and undergoes efficient PTS with its cognate split counterpart.

**Figure 2:**
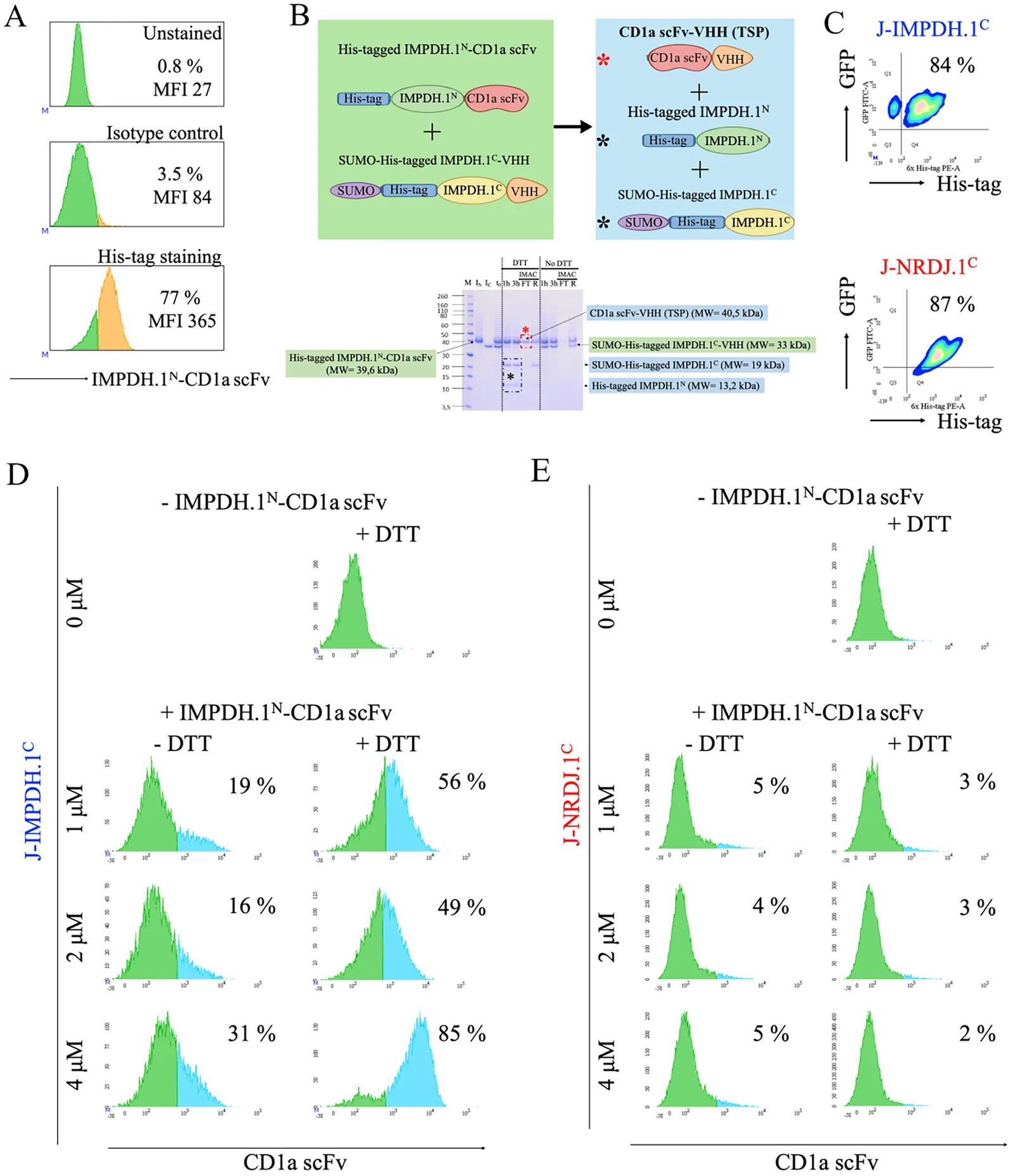
IMPDH.1^N^-CD1a scFv is functional and competent for PTS. **(A)** CD1a^+^ MOLT-4 cells were incubated with IMPDH.1^N^-CD1a scFv, and polypeptide attachment was detected by staining with an anti-His-tag antibody. **(B)** PTS activity of IMPDH.1^N^-CD1a scFv was initially assessed using a model Int^C^ partner designed to transfer a variable heavy domain of a heavy chain (VHH) onto the CD1a scFv upon successful trans-splicing. *Upper:* Schematic representation of the reaction: His-tagged IMPDH.1^N^-CD1a scFv (MW = 39,6 kDa) + small ubiquitin-like modifier (SUMO)-His-IMPDH.1^C^-VHH (MW = 33 kDa) → CD1a scFv-VHH (MW = 40,5 kDa; red asterisk) + His-tagged IMPDH.1^N^ (MW = 13,2 kDa; black asterisk) + SUMO-His-IMPDH.1^C^ (MW = 19 kDa; black asterisk). Reactants (green box); products (blue boxes). Scheme designed with FigureLabs™. To specifically identify the trans-splicing product (TSP), the reaction mixture was applied to a Ni resin (IMAC). Because the TSP lacks a His-tag, it does not bind the resin, like the other reaction components do. *Bottom:* Coomassie-stained SDS-PAGE gel showing reaction products obtained from the PTS assay: I_N_: His-tagged IMPDH.1^N^-CD1a scFv; I_C_: SUMO-His-IMPDH.1^C^-VHH; t_0_: initial time; 1h and 3h refer to the reaction time; FT: flow-through; R: Ni resin-bound proteins. The anticipated molecular weights (MW) are specified in parentheses. **(C)** JURKAT-cells transduced with IMPDH.1^C^-*t*-CAR (J-IMPDH.1^C^, *upper*) or NRDJ.1^C^-*t*-CAR (J-NRDJ.1^C^, *bottom*). Transduction efficacy was determined by GFP reporter and anti-His-tag antibody. (percentage shown in the inset). **(D, E)** JurkaT-cells were used for preliminary technical optimization of the conditions required for split-intein-mediated protein trans-splicing. These experiments were exploratory and intended to identify conditions for subsequent validation in primary human T-cells; therefore, the Jurkat optimization data were not subjected to inferential statistical analysis. Cells were subjected to PTS with IMPDH.1^N^-CD1a scFv under reducing conditions (+DTT). PTS occurred efficiently in J-IMPDH.1^C^ cells **(D)** but not in J-NRDJ.1^C^ cells **(E)**, in a concentration-dependent manner after 3h of incubation. Green denotes the CD1a scFv-negative cell population, whereas cyan blue represents the CD1a scFv-positive population detected using anti-scFv Ab (percentage shown in the inset).

To determine whether the IMPDH.1^N^-CD1a scFv could specifically interact with the membrane-localized IMPDH.1^C^-*t*-CAR, we transduced JURKAT-cells with lentiviral vectors encoding either IMPDH.1^C^-*t*-CAR (**Figure 2C**, upper) or NRDJ.1^C^-*t*-CAR (**Figure 2C**, bottom), the latter as a negative control. Following confirmation of Int^C^-*t*-CAR expression (>84% GFP+), cells were incubated with soluble IMPDH.1^N^-CD1a scFv under both non-reducing and DTT-reducing conditions. In JURKAT-cells expressing IMPDH.1^C^-*t*-CAR, we observed moderate binding of the non-reduced IMPDH.1^N^-CD1a scFv (16-31% of GFP^+^His-tag^+^ cells) that may reflect non-covalent refolding of the split intein fragments or minimal PTS due to partially spontaneously reduced IMPDH.1^N^-CD1a scFv, which provides transient structural stability (**Figure 2D**). Notably, DTT-reduced IMPDH.1^N^-CD1a scFv exhibited largely increased binding to IMPDH.1^C^-*t*-CAR JURKAT-cells (56-86% of GFP^+^His-tag^+^ cells), consistent with an active PTS process. Importantly, binding efficiency increased in a soluble IMPDH.1^N^-CD1a scFv concentration-dependent manner. To confirm the specificity of the interaction, we tested the IMPDH.1^N^-CD1a scFv against JURKAT-cells expressing the orthologous NRDJ.1^C^-*t*-CAR, and no significant binding was observed under either reducing or non-reducing conditions (**Figure 2E**), further confirming the specificity of the IMPDH.1 split inteins in mediating PTS. Collectively, these results indicate that the IMPDH.1 split intein-based PTS platform supports specific and highly efficient PTS under these conditions.

### 2.3 Truncated intein-modified CARs are efficiently expressed in primary T-cells without compromising T-cell expansion, phenotype or exhaustion profile

We next set out to validate our intein-modified CARs in primary human T-cells isolated from healthy donors. Following lentiviral transduction, stable expression of the IMPDH.1^C^-*t*-CAR was observed, as evidenced by the co-expression of GFP and surface localized His-tag. The IMPDH.1^C^-*t*-CAR was detected at least until 12 dpi, with peak surface expression occurring at 5–7 dpi (**Figures 3A, B**). Peak transduction levels (His-tag^+^GFP^+^) reached 15% (**Figure 3B**). We observed a preferential transduction of CD4^+^ T-cells over the CD8^+^ compartment (**Figure 3C**).

**Figure 3:**
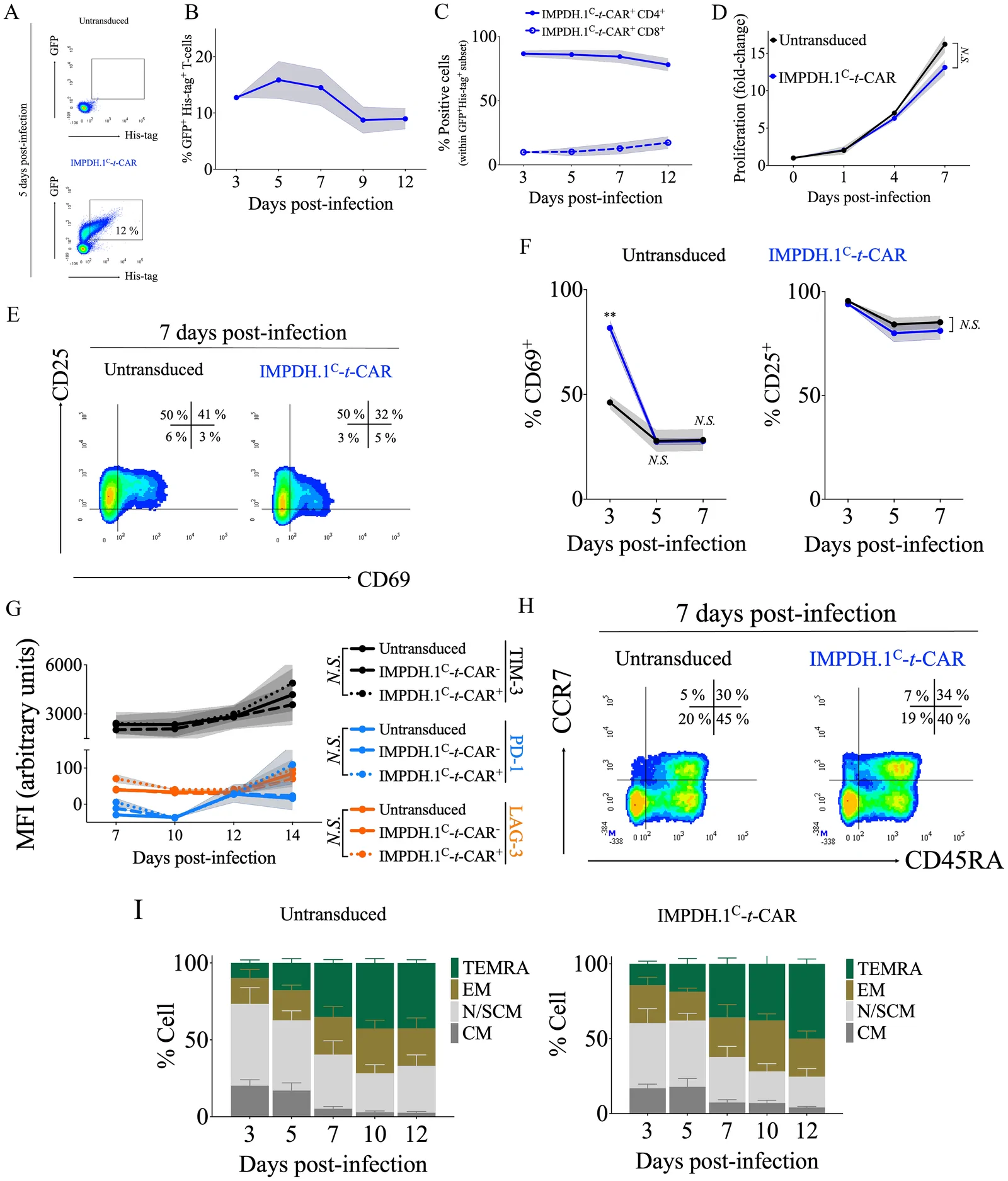
Expression and functional impact of IMPDH^C^-*t*-CAR construct in primary human T-cells. **(A)** Representative expression of IMPDH.1^C^-*t*-CAR in primary human T-cells 5 days post-infection, assessed by GFP and His-tag co-expression. Inset percentage refers to CAR T (GFP^+^His-tag^+^)-cells. **(B)** Quantitative transduction of IMPDH.1^C^-*t*-CAR in primary T-cells shown as the percentage of GFP^+^His-tag^+^ T-cells over time (n=3 donors). **(C)** Kinetics of CD4 and CD8 T-cells within the transduced (GFP^+^His-tag^+^) CAR T-cell population over time (n=3 donors). **(D)** Proliferation of IMPDH.1^C^-*t*-CAR T-cells compared with untransduced controls (n=3 donors). Statistical analyses were performed using two-way ANOVA with Tukey’s multiple-comparisons *post hoc* test. Data are shown as mean ± SEM. Significance levels were defined as *p* < 0.05 (*) and *p* < 0.01 (**); NS denotes not significant. **(E)** Representative analysis of T-cell activation, measured by CD25 and CD69 expression in IMPDH.1^C^-*t*-CAR T-cells 7 days post-infection. **(F)** Temporal evolution of activation status in IMPDH.1^C^-*t*-CAR T-cells as measured by CD69 (*left*) and CD25 (*right*) (n=3 donors). Statistical analyses were performed using two-way ANOVA with Tukey’s multiple-comparisons *post hoc* test. Data are shown as mean ± SEM. Significance levels were defined as *p* < 0.01 (**); NS denotes not significant. **(G)** Evolution of T-cell exhaustion markers over time. IMPDH.1^C^-*t*-CAR T-cells were assessed for TIM-3, PD-1, and LAG-3 expression in *t*-CAR^–^ and *t*-CAR^+^ subsets within the same infected T-cell populations (n=3 donors). Statistical analyses were performed using two-way ANOVA with Tukey’s multiple-comparisons *post hoc* test. Data are shown as mean ± SEM. Significance levels were defined as *p* < 0.05 and *p* < 0.01 (**); NS denotes not significant. **(H)** Representative analysis of memory phenotype distribution in untransduced and IMPDH.1^C^-*t*-CAR T-cells 7 days post-infection. **(I)** Summary of memory phenotype dynamics in untransduced and IMPDH.1^C^-*t*-CAR T-cells (n=3 donors).

IMPDH.1^C^-*t*-CAR T-cells expanded very similarly to non-transduced T-cells (13- *vs.* 16-fold, respectively) over a 7-day expansion period (**Figure 3D**). Kinetics of the T-cell activation markers CD25 and CD69 were not affected by the expression of the IMPDH.1^C^-*t*-CAR (**Figures 3E, F**). Moreover, a 14-day *in vitro* follow-up of the T-cell exhaustion markers TIM-3, LAG-3, and PD-1 revealed no statistically significant differences between IMPDH.1^C^-*t*-CAR-expressing T-cells and untransduced controls (**Figure 3G**). Finally, we observed that expression of IMPDH.1^C^-*t*-CAR did not alter the distribution of naïve/memory/terminally differentiated subsets within the T-cell pool (**Figures 3H, I**). These data indicate that truncated intein-modified CARs are efficiently expressed in primary T-cells without compromising T-cell expansion, activation phenotype or exhaustion profile.

We next investigated whether expression of the truncated IMPDH.1^C^-*t*-CAR receptor affected T-cell biology in the absence of split-intein-mediated CAR assembly and upon T-ALL cell challenge. Exposure of IMPDH.1^C^-*t*-CAR T-cells to CD1a^+^ MOLT-4 cells did not induce detectable antigen-dependent activation, as shown by comparable CD25/CD69 activation profiles to UTD T-cells at both 24 and 72 h (**Supplementary Figures S2A, B**). Consistently, target cell exposure did not significantly alter T-cell memory differentiation or increase the expression of the exhaustion-associated markers PD-1, TIM-3, LAG-3, or TIGIT (**Supplementary Figures S2C, D**). Moreover, IMPDH.1^C^-*t*-CAR T-cells did not restrict MOLT-4 expansion compared with UTD T-cells indicating the absence of cytotoxicity due to basal activation (**Supplementary Figure S2E**). Together, these findings indicate that the truncated receptor remains functionally inert upon encounter with antigen-expressing target cells, with no evidence of tonic or antigen-induced signaling in the absence of conditional CAR assembly.

### 2.4 PTS reconstitutes full-length CARs on the T-cell surface

We previously established the therapeutic potential of CD1a-directed CAR T-cells in controlling T-ALL progression. Building upon these findings, we evaluated the reconstitution of a full-length CD1a-specific CAR utilizing the split intein-mediated platform, as a proof-of-concept. We thus incubated IMPDH.1^C^- or NRDJ.1^C^-*t*-CAR (negative control) T-cells with a DTT-reduced IMPDH.1^N^-CD1a scFv. Flow cytometry analysis, utilizing an APC-conjugated secondary antibody specific for human IgG, confirmed scFv localization on the membrane of IMPDH.1^C^-*t*-CAR T-cells (**Figure 4A**), but not on NRDJ.1^C^-*t*-CAR T-cell controls (**Supplementary Figure S3**) demonstrating IMPDH.1 split intein-specific PTS. It is noteworthy that the 6xHis sequences found in IMPDH.1^N^-CD1a scFv and IMPDH.1^C^-*t*-CAR are excised as components of the split inteins during the reaction process and are not present in the final reconstituted full-length CD1a CAR. We further characterized the efficiency of PTS by examining its dependence on redox conditions and intein pairing. Under non-reducing conditions, the scFv conjugated poorly, with fewer than 15% of the cells showing surface scFv. However, DTT-reduced IMPDH.1^N^-CD1a scFv significantly enhanced the reaction, resulting in 17-66% scFv^+^ cells in the IMPDH.1^C^-*t*-CAR group (**Figures 4A, B**), confirming conditional reconstitution.

**Figure 4:**
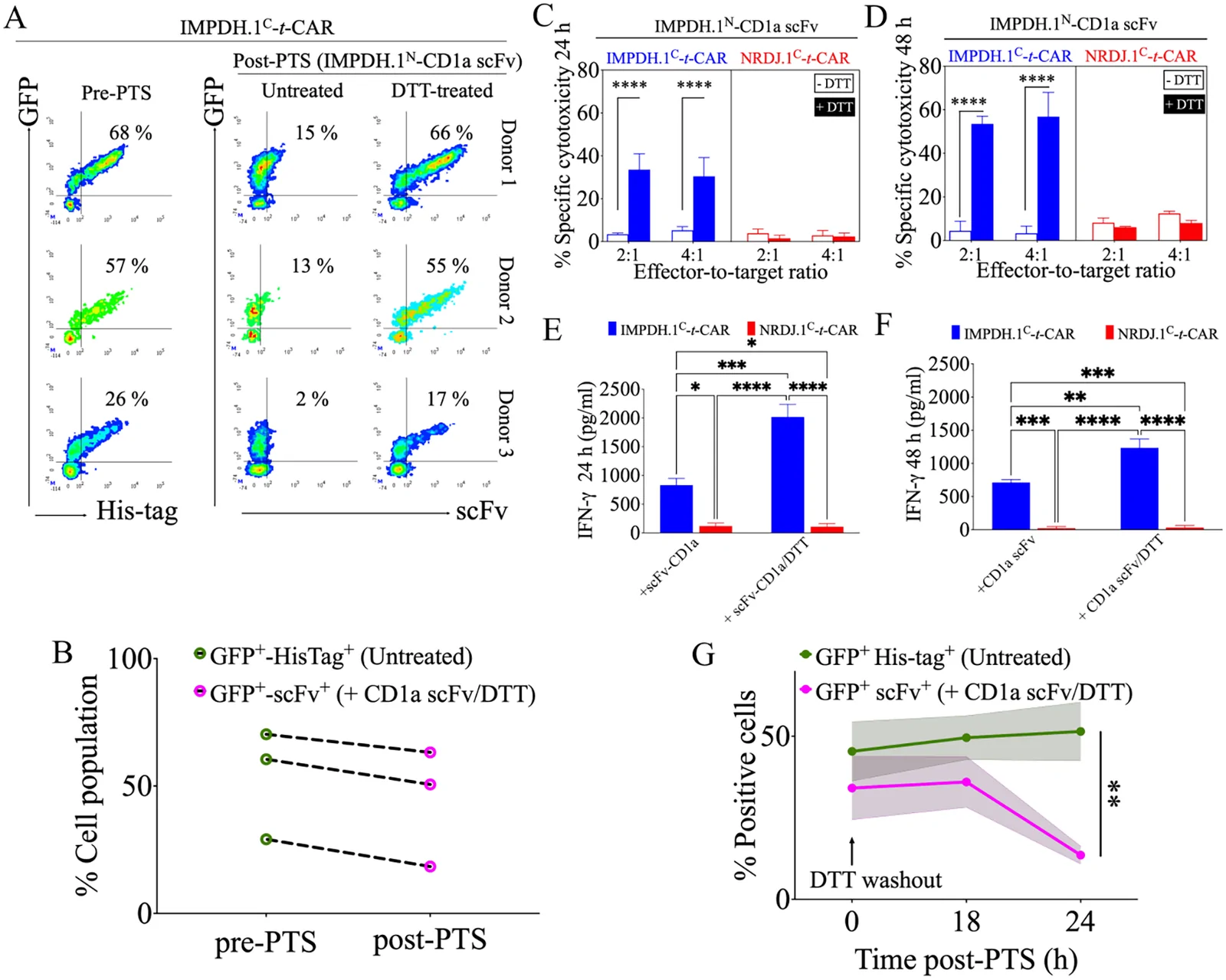
Functional reconstitution of CD1a CAR through IMPDH.1-mediated PTS. **(A)** Primary lymphocytes from three independent donors were transduced with IMPDH.1^C^-*t*-CAR, as identified by GFP^+^His-tag^+^ co-expression (*left*). Cells were then subjected to PTS by incubation with either untreated IMPDH.1^N^-CD1a scFv (*middle*) or DTT-pretreated IMPDH.1^N^-CD1a scFv (*right*). Successful CD1a scFv reconstitution was detected using an anti-scFv antibody. **(B)** Quantification of IMPDH.1^C^-*t*-CAR^+^ T-cells prior to PTS (green circles) and the proportion of reconstituted full-length CD1a CAR^+^ T-cells following PTS with DTT-pretreated IMPDH.1^N^-CD1a scFv (magenta circles) (n=3 donors). **(C, D)** Cytotoxic activity of reconstituted CD1a CAR T-cells generated through IMPDH.1-mediated PTS against CD1a^+^ MOLT-4 cells, measured after 24 h **(C)** and 48 h **(D)** of co-culture, respectively (n=3 donors). DTT-untreated conditions mark as empty columns and DTT-treated counterparts as filled color-columns. **(E, F)** IFN-γ secretion into culture supernatants at 24 h **(E)** and 48 h **(F)** post co-culture with CD1a^+^ MOLT-4 cells, respectively (n=3 donors). **(G)** Analysis of reconstituted CD1a CAR T-cells following DTT washout (*magenta*). As a control, His-tag expression in the corresponding IMPDH.1^C^-*t*-CAR T-cells not subjected to PTS is shown (*green*). Statistical analyses were performed using two-way ANOVA with Tukey’s multiple-comparisons *post hoc* test. Data are shown as mean ± SEM. Significance levels were defined as *p* < 0.05 (*), *p* < 0.01 (**), *p* < 0.001 (***) and *p* < 0.0001 (****); NS denotes not significant.

Importantly, exposure to extracellular PTS-mimicking conditions using a reduced irrelevant protein did not compromise IMPDH.1^C^-*t*-CAR T-cell viability or increase apoptosis (**Supplementary Figure S4**). Furthermore, it did not elicit aberrant activation of either UTD or IMPDH.1^C^-*t*-CAR T-cells, as demonstrated by preserved CD25/CD69 activation profiles (**Supplementary Figure S5**). Collectively, these findings indicate that the extracellular reducing conditions required for split-intein-mediated CAR assembly are well tolerated by primary human T-cells and do not intrinsically perturb their activation state.

### 2.5 Reconstituted CAR T-cells exhibit specific cytotoxicity against T-ALL cells

To evaluate the functional potency of the reconstituted receptors, we performed a cytotoxicity assay. We co-incubated the trans-spliced CAR T-cells with a 1:1 mixture of wild-type CD1a^+^ MOLT-4 and GFP^+^CD1a^-/KO^ MOLT-4 cells, the latter serving as an internal negative control. The survival of CD1a^+^ target cells was monitored by flow cytometry following the gating strategy shown in **Supplementary Figure S6A** and normalized against the GFP^+^CD1a^-/KO^ population. Following 24 and 48 hours of co-culture, a significant reduction in GFP^-^CD1a^+^ MOLT-4 cells was observed only in the group with IMPDH.1^C^-*t*-CAR T-cells treated with DTT-reduced IMPDH.1^N^-CD1a scFv (**Figures 4C, D**, **Supplementary Figures S6B, C**). By contrast, no significant cytotoxicity was observed in the NRDJ.1^C^-*t*-CAR control group or in cells treated with non-reduced scFv, confirming that T-cell lysis is strictly dependent on both the specific split intein pairing and successful redox-mediated PTS. To corroborate these findings, we quantified IFN-γ secretion in culture supernatants. Consistent with the cytotoxicity assays, only IMPDH.1^C^-*t*-CAR T-cells subjected to effective PTS exhibited significantly elevated IFN-γ levels at both 24 and 48 hours upon encountering CD1a^+^ T-ALL cells (**Figures 4E, F**), consistent with antigen-specific T-cell activation.

### 2.6 Reconstituted CAR expression declines over time post-PTS

To evaluate the stability of the reconstituted CAR, we monitored scFv surface expression upon removal of unbound scFv and DTT. Both the frequency of scFv^+^ cells and the mean fluorescence intensity declined within 24 hours, indicating the natural turnover of the reconstituted full-length CAR and the subsequent reversion to truncated CAR expression (**Figure 4G**). Importantly, the underlying IMPDH.1^C^-*t*-CAR scaffold is replenished by ongoing cellular translation following consumption in the PTS reaction (**Figure 4G**). This suggests that the T-cells remain “primed” for repeated PTS events, enabling sustained CD1a-specific targeting or the sequential retargeting of alternative antigens by introducing different Int^N^-scFv fusions.

## 3 Discussion

Split intein-mediated CAR T-cell systems represent a strategy to regulate receptor assembly at the protein level. In this study, we demonstrate that membrane-anchored C-terminal fragments (IMPDH.1^C^) can be reconstituted into functional CARs through PTS upon exposure to soluble N-terminal (IMPDH.1^N^) scFv fragments. This approach enables conditional receptor formation and results in antigen-specific cytotoxic activity against CD1a^+^ leukemia cells. Unlike earlier studies on split-intein-based modular CAR systems (referred to as CARtein), which primarily investigated multitargeting functions (12), the current research focuses on the mechanistic and biological aspects of conditional split intein-mediated CAR assembly in primary human T-cells. This included examining impacts on T-cell phenotype, exhaustion status, and transient receptor reconstitution dynamics.

Importantly, expression of the truncated intein-modified CAR scaffold did not compromise T-cell expansion, activation, exhaustion or memory subset distribution under the conditions tested. The absence of altered CD25/CD69 expression, exhaustion marker induction (TIM-3, PD-1, LAG-3), or impaired T-cell expansion suggests that the truncated scaffold does not induce detectable tonic siganlling.

The higher frequency of transduced cells observed within the CD4^+^ compartment may reflect subset-specific differences in permissiveness to lentiviral gene transfer. Differential activation and proliferative responses, memory-subset composition, and expression of intrinsic antiviral restriction factors between CD4^+^ and CD8^+^ T-cells can influence lentiviral entry and post-entry processing, potentially favoring transduction and/or transgene expression in CD4^+^ T-cells under the culture conditions used here. Importantly, such CD4/CD8 differences have been reported previously for lentiviral gene transfer and appear to be dependent on the vector and T-cell activation conditions rather than representing an invariant property of either T-cell subset (20–22).

Following PTS-mediated reconstitution, CAR T-cells exhibited antigen-specific cytotoxicity and cytokine secretion. However, surface exposure of the reconstituted CAR declined over time, suggesting that receptor assembly is transient in this system. The implications of this transient behavior for therapeutic efficacy upon multiple PTS remain to be determined.

A distinctive feature of this strategy is the physical separation of antigen recognition from the membrane-anchored CAR signaling machinery, enabling receptor assembly at the protein rather than the genetic level. In the present proof-of-concept, we deliberately employed a previously validated CD1a-targeting architecture to establish the feasibility and functional consequences of split-intein-mediated extracellular CAR reconstitution while minimizing variability associated with changes in antigen or binder properties. Thus, although our findings establish the modular architecture of the system, they do not yet demonstrate unrestricted interchangeability of antigen-recognition modules. Extending this approach to additional antigens and binding domains will therefore be important to establish its generalizability and to determine whether targeting modules can be exchanged while retaining efficient protein trans-splicing and CAR function.

Although not directly evaluated in the present study, split intein-mediated CAR reconstitution could provide a framework for conditional post-translational regulation of CAR activity. Future studies should investigate whether this approach enables temporal control of receptor function, transient activation of engineered T-cells, and modulation of CAR signaling dynamics. In addition, it will be important to determine whether regulated CAR assembly can reduce tonic signaling or mitigate fratricide-associated effects in T-cell malignancies, where constitutive CAR expression may adversely affect T-cell fitness and therapeutic performance.

Whereas the ProtinCAR platform developed by Jiang Y et al. utilizes a chemically modified adapter—specifically, a C-terminal split intein sequence fused to an anti-CD19 antibody—our approach involves producing the soluble intein-scFv adaptor as a recombinant, secreted protein in mammalian CHO cells (16). This method facilitates recovery via standard biologics manufacturing processes and ensures preservation of native folding and post-translational modifications.

The results presented here establish the feasibility of split intein-mediated CAR reconstitution in primary human T-cells. The observed transient expression of the reconstituted receptor and the persistence of the membrane-anchored scaffold suggest that controlled receptor assembly may provide a means to modulate CAR availability at the cell surface. This control may help mitigate possible toxicities arising from sustained T cell activation such as the cytokine release syndrome (23, 24). Future studies will be required to determine whether this approach can support repeated rounds of receptor reconstitution, enable antigen retargeting through alternative adaptor molecules, or permit quantitative control of receptor abundance and function. In addition, the effects of this strategy on CAR T-cell persistence, safety, and therapeutic efficacy remain to be evaluated in relevant *in vivo* models.

### 3.1 Limitations and future directions

While our findings establish the feasibility of split intein–mediated CAR reconstitution, several challenges remain. First, although DTT was effective *in vitro* for reducing scFv thiol groups to enable splicing, its clinical utility is limited due to plausible toxicity and lack of specificity. Future work should focus on developing biocompatible reducing agents or redox-independent systems to trigger PTS *in vivo*.

Second, the *in vivo* persistence and functional longevity of trans–spliced CAR T-cells remain unknown. Given the observed decay in scFv expression, repeated administrations of scFv or engineering more stable intein pairs may be necessary to sustain therapeutic activity over time. Future studies will be required to optimize the pharmacokinetic properties of the Int^N^-scFv adaptor molecules and evaluate how such modifications affect biological activity *in vivo*.

Finally, although the use of His-tags enabled effective experimental detection and quantitative analysis in this proof-of-concept study, future versions aimed at clinical applications will likely utilize tag-free constructs to minimize the risk of immunogenicity arising from non-native peptide sequences.

In summary, our findings establish the feasibility of split intein-mediated CAR reconstitution in primary human T-cells and provide a foundation for further development of post-translational strategies to regulate CAR T-cell activity.

## 4 Materials and methods

### 4.1 Reagents, drugs, and antibodies

Advanced DMEM, RPMI-1640, L-glutamine, and penicillin/streptomycin (P/S) were purchased from GIBCO/Invitrogen (Waltham, MA). Phosphate-buffered saline (PBS) was obtained from Merck Life Science (Darmstadt, Germany). Human (h) IL-7 and hIL-15 were purchased from Miltenyi Biotec (Bergisch Gladbach, Germany). Fetal bovine serum (FBS) was purchased from Sigma-Aldrich (St. Louis, MO). Anti-CD3 (OKT3) and anti-CD28 (CD28.2) monoclonal antibodies (mAbs), and phycoerythrin (PE)-, allophycocyanin (APC)-, Brilliant Violet 421 (BV421)-, and BV510-conjugated mAbs specific for human CD3 (SK7), CD1a (HI149), CD25 (M-A251), CCR7 (150503), CD45RA (5H9), LAG-3 (T47-530), TIM-3 (7D3), PD-1 (MIH4), CD69 (FN50), anti-His (J095G46), and isotype-matched negative control mAbs, were purchased from BD Biosciences (Franklin Lakes, NJ). Allophycocyanin (APC) AffiniPure^®^ F(ab’)_2_ Fragment Goat Anti-Human IgG (H+L) (polyclonal, 109-136-088) was purchased from Jakson ImmuoResearch. CellTrace™ Violet reagent (C34557) and dithiothreitol (DTT, R0861) were purchased from ThermoFisher Scientific (Danvers, MA).

### 4.2 Design and production of Int^N^-CD1a scFv constructs

The scFv recognizing CD1a (17) was genetically fused to the N-terminus of the IMPDH.1^N^ intein fragment. For detection and purification purposes, a C-terminal His-tag was included at the C-terminus, while an IgK signal peptide was added to the N-terminus to promote protein secretion. Both constructs were expressed in TurboCHO cells using GenScript proprietary trasnfection reagents (For technical details check GenScript, Rijswijk, The Netherlands). The IMPDH.1^N^-CD1a scFv fusion proteins were purified by immobilized metal affinity chromatography using a 5-mL Ni column connected to an ÄKTA chromatography system (Cytiva, Marlborough, MA). Eluted fractions were analyzed by SDS-PAGE followed by Coomassie blue staining. Fractions containing the target proteins were pooled and dialyzed extensively against PBS. Protein concentrations were determined spectrophotometrically, and purified samples were stored at -80 °C until use.

### 4.3 Protein and protein complex prediction

Protein structure predictions were generated using AlphaFold v3 with default parameters. The full-length amino acid sequences of the target proteins (IMPDH.1^N^-CD1a and IMPDH.1^C^-*t*-CAR) was used as input. Multiple sequence alignments and structural inference were performed using the standard AlphaFold v3 pipeline and reference databases. Five candidate models were generated, and the top-ranked model was selected for downstream analyses based on the predicted Local Distance Difference Test (pLDDT) confidence score. Model reliability was assessed using per-residue pLDDT values and the predicted aligned error matrix when applicable. The top-ranked prediction was used for structural visualization. Structural visualization and figure preparation were performed using ChimeraX v1.10 (University of California, San Francisco).

### 4.4 Validation of trans-splicing activity

The trans-splicing activity of purified IMPDH.1^N^-CD1a fusions was evaluated using a model IMPDH.1^C^-POI (protein of interest) construct containing the complementary IMPDH.1^C^ fragment. Reactions were carried out in PBS, with the IMPDH.1^C^-POI fusion added at a two-fold molar excess relative to the IMPDH.1^N^-CD1a fusion. Reactions were incubated at 25 °C in the presence or absence of a reducing agent (DTT). Aliquots were collected at 1, 2, and 4 hours after initiation to monitor reaction progress. Samples were analyzed by SDS-PAGE followed by Coomassie staining to assess the efficiency of the trans-splicing reaction. In the case of the IMPDH.1^N^-CD1a fusion, the reaction mixture remaining after 4 hours of incubation was subsequently loaded onto a Ni-resin column to unambiguously confirm the formation of the trans-splicing product. The resin was expected to retain all reaction components except for the trans-splicing product, as this was the only molecule lacking a His-tag.

### 4.5 Cell lines

The human T-ALL cell lines JURKAT and MOLT-4 cells were purchased from the DSMZ cell line bank (Braunschweig, Germany) and cultured in RPMI-1640 supplemented with 10% heat-inactivated FBS. GFP^+^ CD1a *knock-out* (KO) MOLT-4 cells were generated by CRISPR-mediated genome editing and reported elsewhere (17). Briefly, half-million cells were electroporated using the Neon Transfection System (ThermoFisher Scientific) with a Cas9/crRNA:tracrRNA complex (IDT). The following crRNA guide was designed for the *CD1A* gene: 5’-TATTCCGTATACGCACCATTCGG-3’. After electroporation, cells were allowed to recover, and the different KO clones or populations expressing varying levels of CD1a were sorted by flow cytometry.

### 4.6 Lentiviral construct design and production

The truncated CAR construct (Int^C^-*t*-CAR) was generated by fusing either the IMPDH.1^C^ or NRDJ.1^C^ (negative control) split intein fragment to a signaling scaffold comprising a CD8 hinge, a CD8α transmembrane domain, and the 4-1BB and CD3ζ intracellular signaling domains. A T2A sequence was used to co-express green fluorescent protein (GFP) as a marker. A 6× His-tag was added at the extracellular N-terminus of the Int^C^-*t*-CAR to allow quantification. The construct was cloned into the clinically validated second-generation lentiviral backbone pCCL. A control lentiviral vector expressing GFP alone (MOCK) was also prepared. Lentiviral particles were produced by polyethyleneimine-mediated transfection of HEK293T-cells with plasmids encoding pRRE, pREV, pVSV-G, and the CAR construct (Polysciences Inc., Warrington, PA). Viral supernatants were harvested 72 hours later and concentrated by ultracentrifugation.

Functional lentiviral vector (LV) titers were determined by transduction of HEK293T-cells and quantification of GFP expression by flow cytometry. HEK293T-cells were seeded in 6-well plates at 2,5×10^5^ cells/well 24 hours before transduction. Serial dilutions of LV supernatant were prepared in DMEM supplemented with 8 µg/mL polybrene and added to the cells. Cells were cultured for an additional 48 hours to allow transgene expression. The percentage of GFP^+^ cells was measured by flow cytometry. Viral titers were calculated using dilutions yielding <20-30% GFP^+^ cells to minimize the probability of multiple integrations. Functional titers were expressed as transducing units per milliliter (TU/mL) according to the following formula:


TU (mL)=[N x P x D]/V 

where: N=number of target T-cells at the time of transduction; P= fraction of GFP^+^ cells; D=dilution factor of the viral sample; V=volume of diluted virus added to the well, in mL.

### 4.7 T-cell isolation, activation, and transduction

Peripheral blood mononuclear cells (PBMCs) were isolated from healthy donor buffy coats, obtained from the Barcelona Blood and Tissue Bank (BST) under Institutional Review Board approval (#HCB/2018/0030) *via* Ficoll-Hypaque (ThermoFisher Scientific) density gradient centrifugation. Primary T-cells were activated on OKT3/CD28 Abs-coated plates in the presence of hIL-7 and hIL-15 (10 ng/mL each). Following 48 hours of activation, the T-cells were transduced with Int^C^-*t*-CAR lentiviral particles at a multiplicity of infection of 10. Cultures were maintained in RPMI-1640 medium with 10% FBS, 2 mM L-glutamine, and antibiotics. Transduction efficiency and CAR expression were monitored by GFP fluorescence and anti-His-tag staining using flow cytometry.

### 4.8 T-cell immunophenotyping and activation markers

To assess the T-cell memory phenotype, cells were stained with antibodies against CCR7 and CD45RA, allowing classification into naïve/stem central memory (CCR7^+^CD45RA^+^), central memory (CCR7^+^CD45RA^−^), effector memory (CCR7^−^CD45RA^−^), and terminally differentiated effector memory (CCR7^−^CD45RA^+^) subsets. T-cell activation was assessed using CD25 and CD69 surface expression at 3, 5, 7, 10 and 12 days post-infection (dpi).

### 4.9 Protein trans-splicing assay

IMPDH.1^N^-CD1a scFv was reduced by incubation with 5 mM DTT for 1 hour at RT in PBS1X. Following reduction, the protein solution was diluted in complete RPMI medium to achieve a final IMPDH.1^N^-CD1a scFv concentration of 2 μM. Then, T-cells expressing IMPDH.1^C^-*t*-CAR or NRDJ.1^C^-*t*-CAR in complete RPMI medium were added to the reduced IMPDH.1^N^-CD1a scFv and incubated for 3 hours. The cells were then washed with fresh RPMI-1640 supplemented with 10% heat-inactivated FBS to quench remaining DTT and stained with an APC-conjugated secondary antibody specific for human IgG (H+L) to detect scFv conjugation to the cell surface. The PTS efficiency was calculated as the percentage of scFv^+^ cells relative to the initial His-tag^+^ population. Specificity was evaluated using the ortholog NRDJ.1^C^ intein as a negative control. Alternatively, CAR T-cells were incubated with target cells after PTS to measure cytotoxicity.

### 4.10 Cytotoxicity assay against CD1a^+^ T-ALL cells

To assess CAR T-cell cytotoxicity, a 1:1 mixture of wild-type CD1a^+^ (**Supplementary Figure S1**) and CD1a KO MOLT-4 cells was prepared. The MOLT-4 cell mixture was stained with CellTrace Violet reagent and co-cultured with trans-spliced CAR T-cells at an effector (GFP^+^ T-cells): target (E:T) ratio of 2:1 and 4:1 for 24 or 48 hours. Cell viability was assessed by flow cytometry based on the percentage of GFP^-^ cells within the Cell Violet^+^ population (**Supplementary Figure S6A**). Secreted IFN-γ levels were quantified by ELISA (R&D Systems, Minneapolis, MN). Cytotoxicity was calculated as follows


$$Specific\;cytotoxicity\;(\%)=100-(\%\;wt_{MOLT4}sample)/\;(\%\;wt_{MOLT4}PBS)$$

### 4.11 Full-length CAR decay analysis

Following PTS-mediated reconstitution, T-cells were washed to remove residual DTT and unbound scFv fragments, then returned to culture for 24 h. Surface scFv expression was monitored by flow cytometry at 0, 18, and 24 h to assess the stability of the reconstituted CAR.

### 4.12 Statistics

Data are presented as mean ± SEM. Statistical analyses were performed using GraphPad Prism version 8.0 (GraphPad Software, San Diego, CA, USA). Comparisons among multiple groups were conducted using one-way ANOVA followed by Tukey’s multiple-comparison test, unless otherwise indicated. For grouped datasets, two-way repeated-measures ANOVA followed by Šídák’s multiple-comparison test was used. The statistical methods applied to each dataset are specified in the corresponding figure legends, as appropriate.

## Data Availability

The original contributions presented in the study are included in the article/**Supplementary Material**. Further inquiries can be directed to the corresponding authors.
