# Supplementary material for: Split intein-mediated reconstitution of regulatable CAR T-cells
Source: Front Immunol. 2026 Oct 2;17:1844972. doi: 10.3389/fimmu.2026.1844972 (PMC13672617; doi:10.3389/fimmu.2026.1844972)
Supplement: Supplementary file 1 [file DataSheet1.pdf]

Supplementary information

Split intein-mediated reconstitution of regulatable CAR T-cells

SI1. Supplementary tables.

Table S1. AlphaFold 3 confidence metrics and cross-model interface consistency of the predicted split-intein complex.

Metric	CD1a–scFv/IMPDH
Top-ranked model	0
ipTM	0.49
Ranking score	0.68
Mean contact-pair PAE across models	3.86–4.64 Å
Mean antigen-interface Jaccard	0.861
Antigen-interface Jaccard range Mean	0.800–0.915
scFv/partner-interface Jaccard scFv/	0.904
partner-interface Jaccard range Mean	0.874–0.918
residue-pair contact-map Jaccard	0.785
Contact-map Jaccard range	0.697–0.836

Note: Five structural models were generated using AlphaFold 3 (AF3). ipTM and ranking scores correspond to native AF3 confidence metrics, whereas interface Jaccard indices, residue-pair contact-map Jaccard indices, and contact-specific predicted aligned error (PAE) were calculated by downstream structural analysis. Inter-chain contacts were defined as residue pairs containing at least one non-hydrogen atom within 5 Å of the opposing chain. Jaccard similarity coefficients were calculated across all 10 pairwise comparisons among the five AF3 models, with values approaching 1 indicating greater conservation of the predicted interface. Contact-specific PAE represents the mean bidirectional PAE of predicted inter-chain residue contacts, with lower values indicating greater confidence in their relative positioning. Derived interface metrics are not native AF3 confidence scores.

SI2. Supplementary figures.

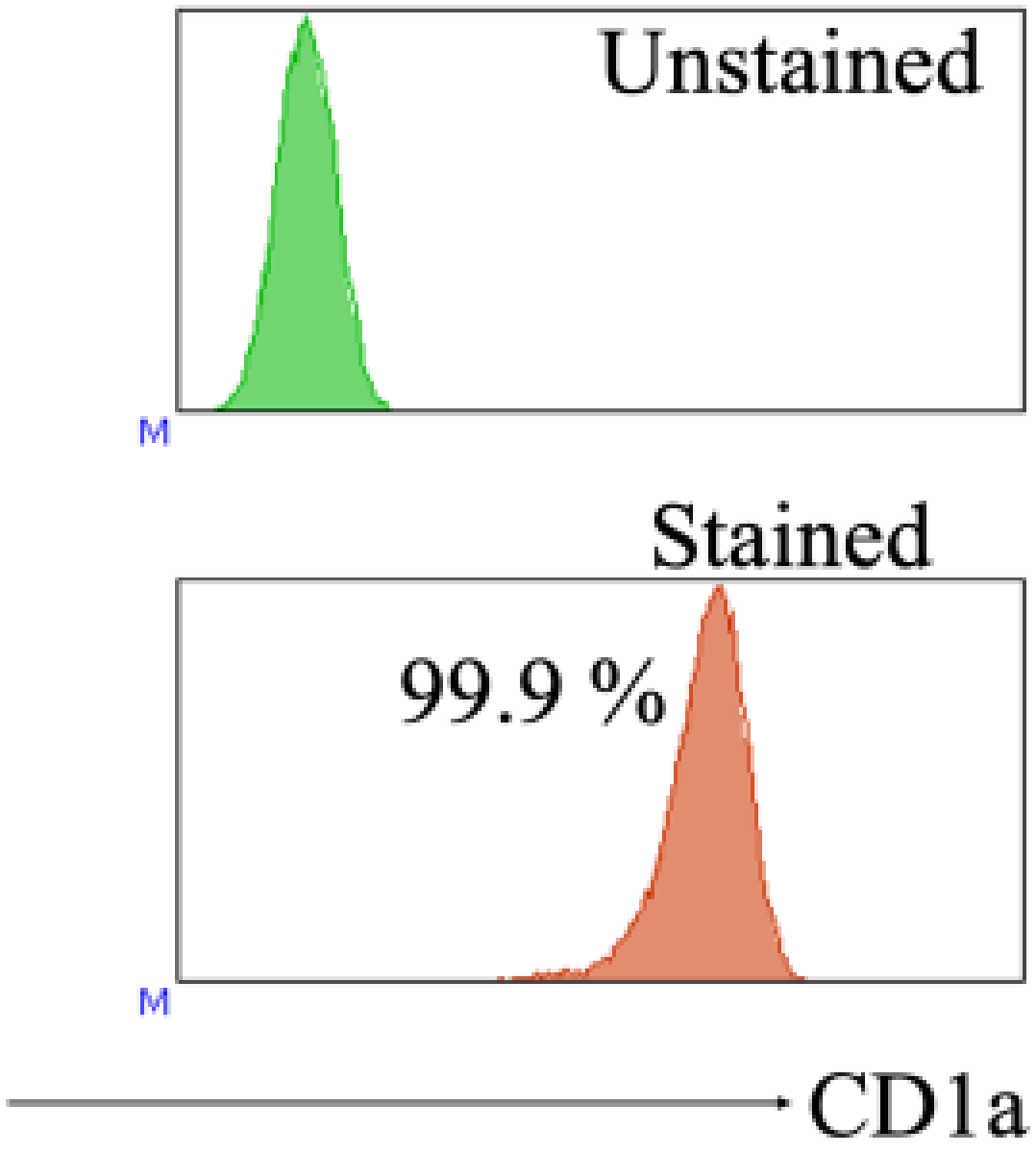

Figure S1. The T-ALL cell line MOLT-4 expresses CD1a.

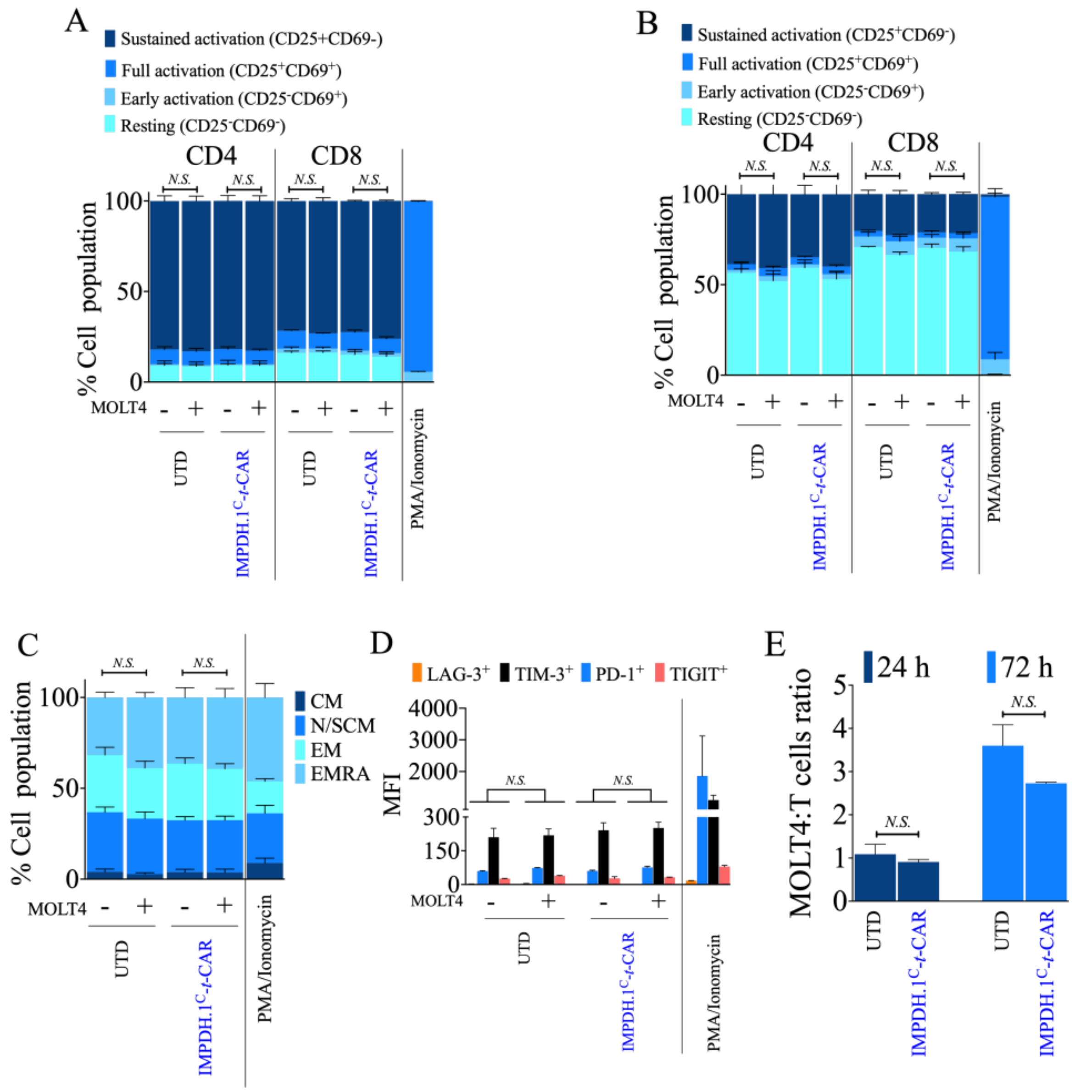

Figure S2. Encounter with target cells does not trigger tonic signalling and activation of IMPDH.1^{C}-*t*-CAR T-cells. Activation phenotype of $CD4^+$ and $CD8^+$ T-cells cultured in the absence or presence of MOLT-4 cells after (A) 24 h and (B) 72 h of co-incubation. Activation status was determined by CD25 and CD69 expression and classified as resting ($CD25^-CD69^-$), early activated ($CD25^-CD69^+$), fully activated ($CD25^+CD69^+$), and sustained activated ($CD25^+CD69^-$). PMA/ionomycin stimulation was included as a positive control. (C) Memory differentiation profile of T-cells after co-culture with MOLT-4 cells for 72 h. T-cell subsets were identified according to CD45RA and CCR7 expression as naïve/stem cell memory (N/SCM; $CD45RA^+CCR7^+$), central memory (CM; $CD45RA^-CCR7^+$), effector memory (EM; $CD45RA^-CCR7^-$), and terminally differentiated effector memory (EMRA; $CD45RA^+CCR7^-$). PMA/ionomycin-treated cells served as a positive activation control. (D) Surface expression of the exhaustion markers PD-1, TIM-3, LAG-3, and TIGIT in UTD and IMPDH.1^{C}-*t*-CAR T-cells following co-culture with MOLT-4 target cells for 72 h. Marker expression was quantified as mean fluorescence intensity (MFI). (E) Expansion of MOLT-4 cells during co-culture with UTD or IMPDH.1^{C}-*t*-CAR T-cells, expressed as the MOLT-4: T-cell ratio after 24 h and 72 h. Data are presented as the mean ± SEM of three

independent healthy donors. Statistical analyses were performed using two-way repeated-measures ANOVA followed by Šídák's multiple-comparison test. N.S., not significant.

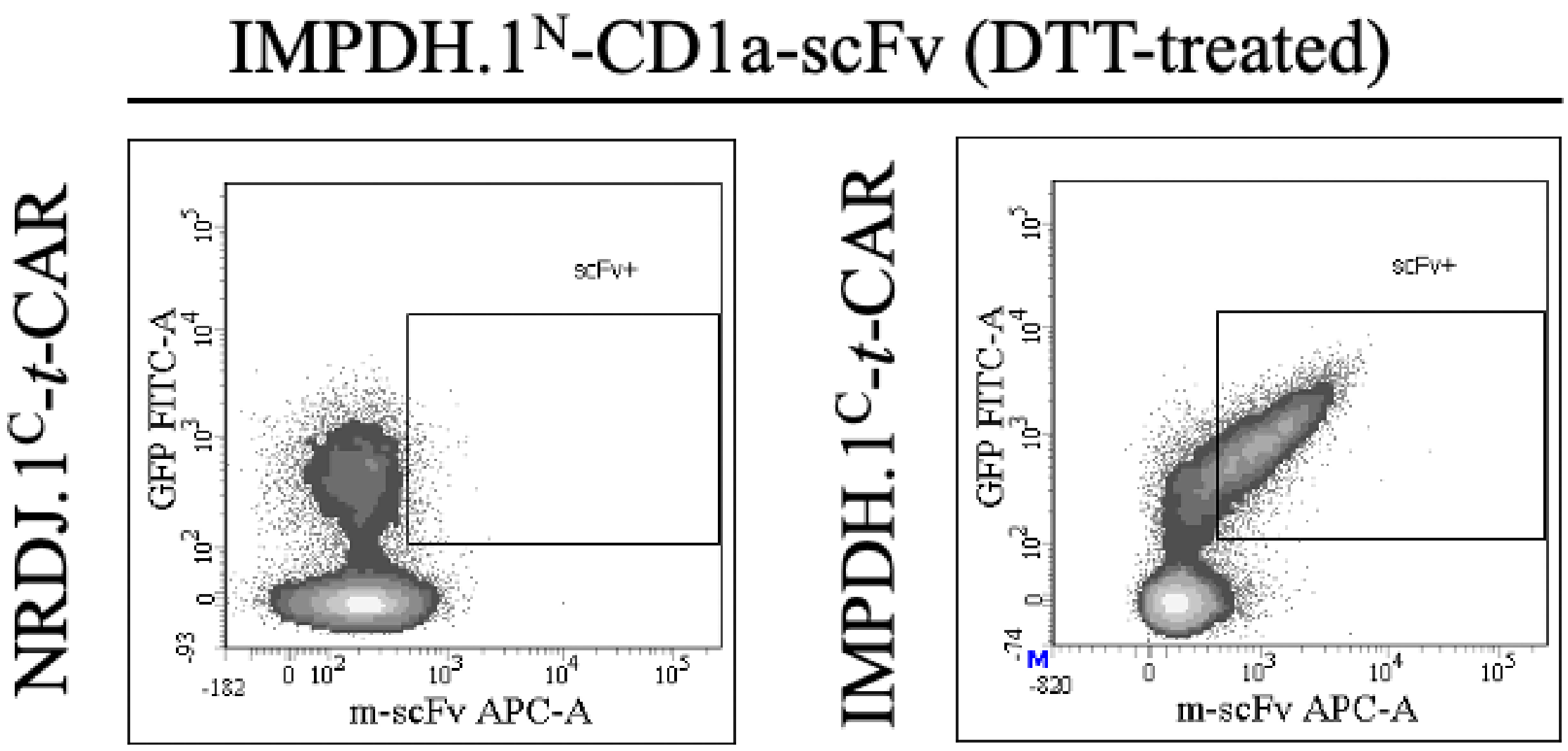

Figure S3. Representative analysis of primary lymphocytes from a healthy donor transduced with Nrdj.1^{C}-*t*-CAR (*left*) or IMPDH.1^{C}-*t*-CAR (*right*) and subjected to PTS by incubation with DTT-pretreated IMPDH.1^{N}-CD1a scFv for 3h. Successful CD1a scFv reconstitution was detected using an anti-scFv antibody. The x-axis refers to scFv.

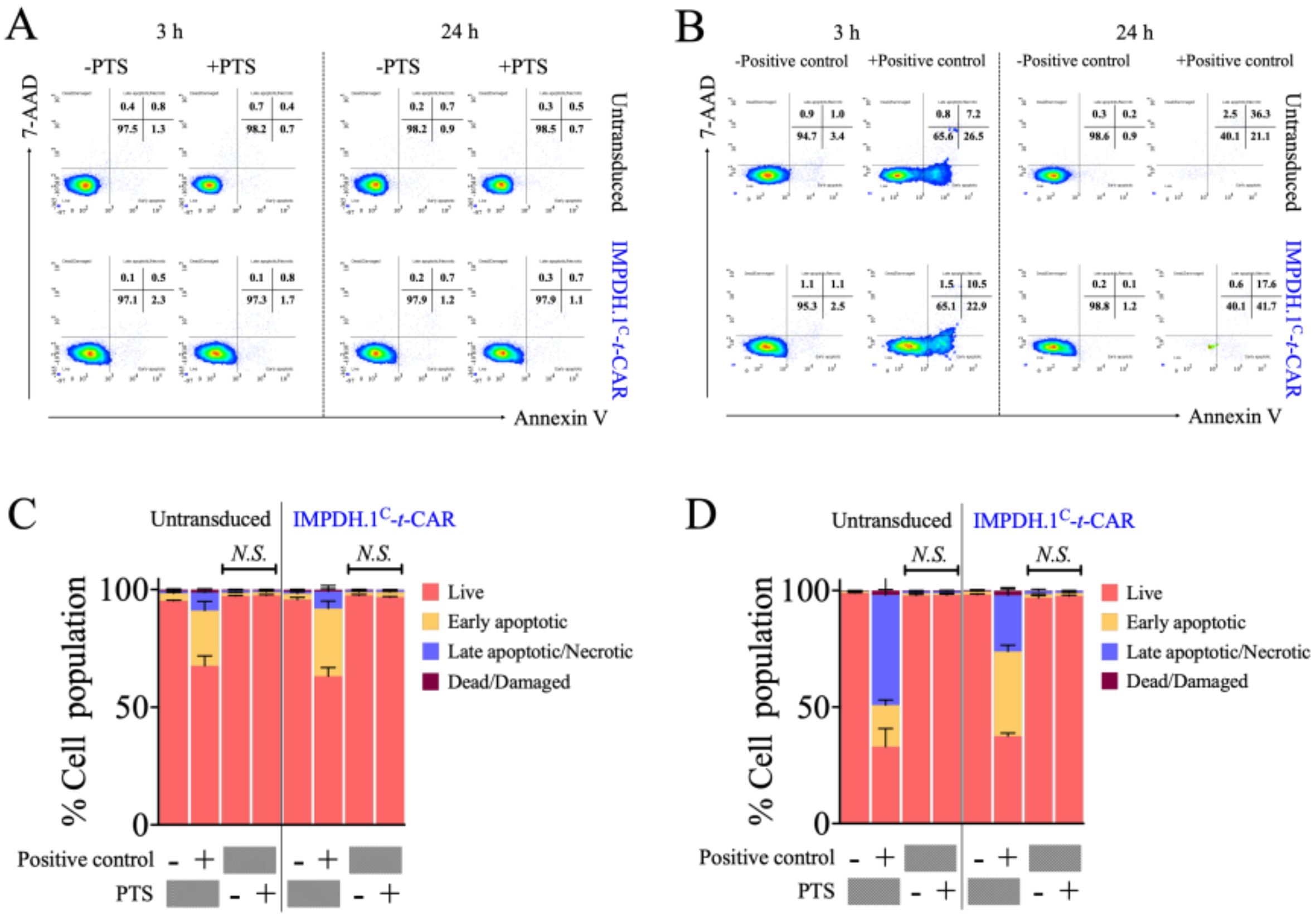

Figure S4. PTS under reducing conditions do not induce apoptosis of IMPDH.1^{C}-*t*-CAR T-cells. Apoptosis levels at 3 and 24 h assessed by Annexin V and 7-AAD staining, (A) under (PTS)-mimicking conditions and (B) 2.5 mM DTT treatment (positive control). Live (Annexin V^{-}/7-AAD^{-}), Early apoptotic (Annexin V^{+}/7-AAD^{-}), Late apoptotic/Necrotic (Annexin V^{+}/7-AAD^{+}), or Dead/Damaged (Annexin V^{-}/7-AAD^{+}). Representative dot plots from one donor are shown. Summary of apoptosis level at (C) 3 h and (D) 24 h. Bar graphs represent the mean ± SEM of three independent donors. Statistical analyses were performed using two-way repeated-measures ANOVA followed by Šídák's multiple-comparison test. *N.S.*, not significant.

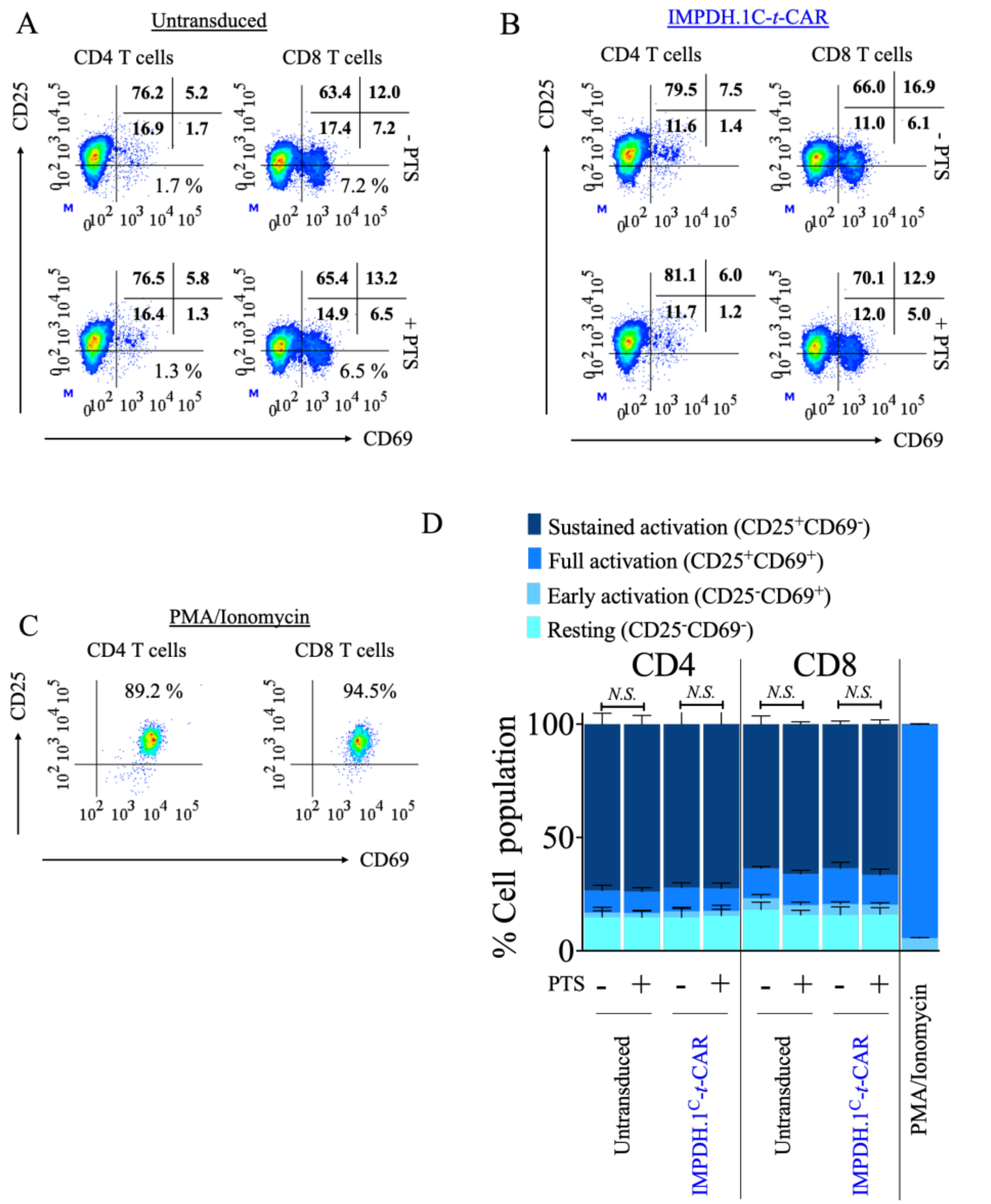

Figure S5. PTS under reducing conditions does not induce activation of untransduced or IMPDH.1^{C}-*t*-CAR T-cells. Representative flow cytometry plots showing CD25 and CD69 expression on $CD4^{+}$ and $CD8^{+}$ (A) untransduced and (B) IMPDH.1^{C}-*t*-CAR T-cells following incubation with or without DTT-reduced irrelevant protein (PTS) for 24 h. Cells were classified into four activation subsets based on CD25 and CD69 expression: resting ($CD25^{-}CD69^{-}$), early activated ($CD25^{-}CD69^{+}$), sustained activation ($CD25^{+}CD69^{-}$), and fully activated ($CD25^{+}CD69^{+}$). (C) Representative CD25/CD69 staining of PMA/Ionomycin-treated T-cells used as a positive control for activation. (D) Quantification of activation subsets in $CD4^{+}$ and $CD8^{+}$ untransduced and IMPDH.1^{C}-*t*-CAR T-cells. Bars represent the mean percentage of cells within each activation subset from three independent

donors, with error bars indicating SEM. Statistical analysis was performed using a two-way repeated-measures ANOVA followed by Šídák's multiple-comparison test.

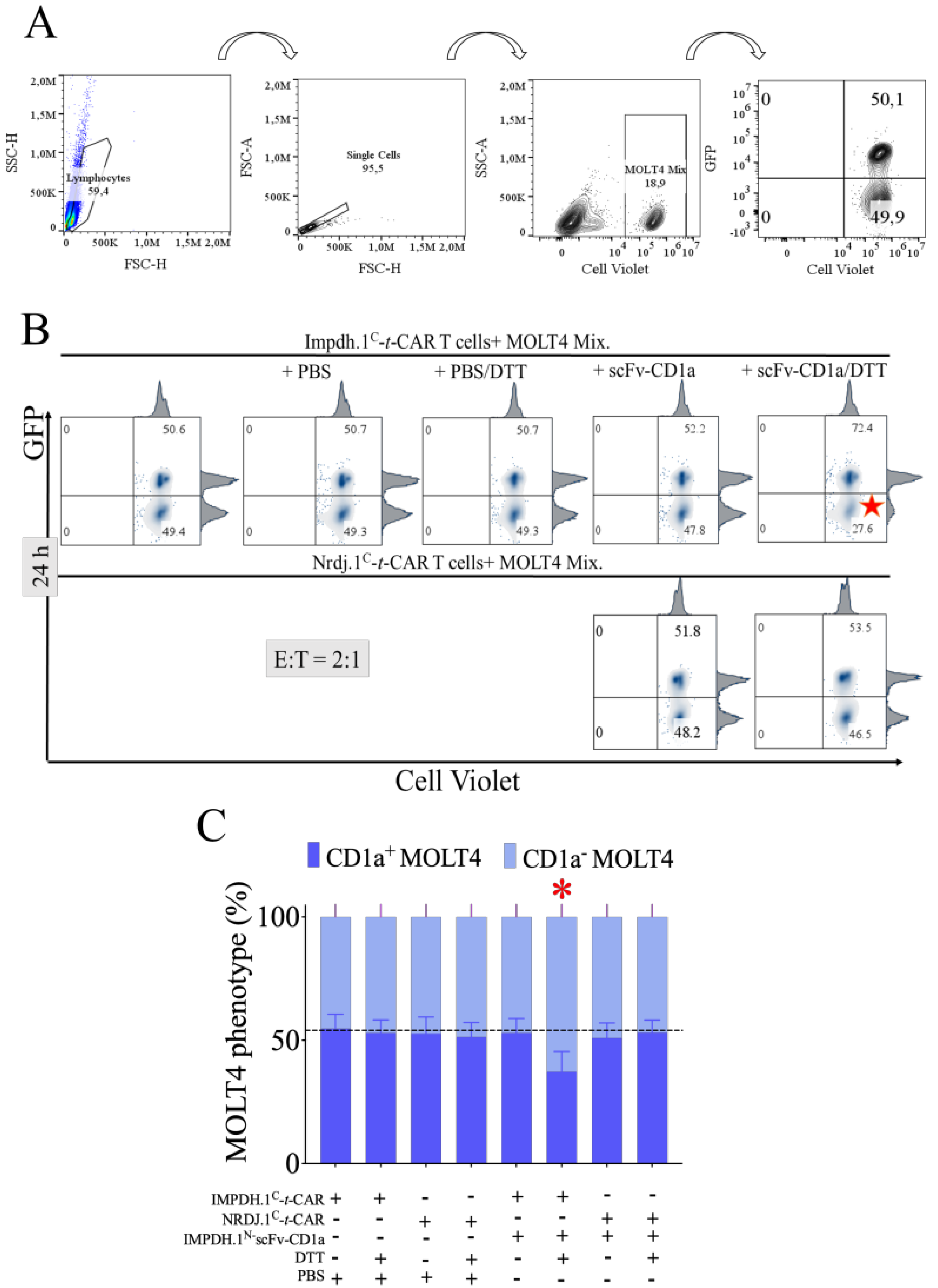

Figure S6. Cytotoxic activity of effector T-cells against CD1a+ MOLT-4 cells. CD1a wild-type and CD1a-*KO* MOLT-4 cells (the latter engineered to express GFP) were mixed at a 1:1 ratio, stained with Cell Violet, and co-cultured with the indicated effector T-cells. (A) Gating strategy used to identify target MOLT-4 cells, defined as

Cell Violet^{+} either CD1a^{+} (GFP^{-}) or CD1a$^{-/KO}$ (GFP^{+}). (B) Representative example from one T-cell donor after 24 h of co-culture at an E:T ratio of 2:1. Red star marks MOLT-4 cells co-cultured with IMPDH.1-mediated, but not NRDJ.1-mediated, CD1a CAR-reconstituted T-cells, illustrating the evident reduction of CD1a^{+} MOLT-4 target cells. Baseline cytotoxicity was assessed using the following control conditions: IMPDH.1^{C}-*t*-CAR T-cells challenged with the MOLT-4 mixture; IMPDH.1^{C}-*t*-CAR T-cells pretreated with PBS and challenged with the MOLT-4 mixture; and, IMPDH.1^{C}-*t*-CAR T-cells pretreated with PBS/5 mM DTT and challenged with the MOLT-4 mixture. (C) Quantification of CD1a^{+} (GFP^{-}) or CD1a$^{-/KO}$ (GFP^{+}) MOLT-4 cell proportions following 24 h of co-culture at an E:T ratio of 2:1 (n=3 donors). Red asterisk marks MOLT-4 cells co-cultured with IMPDH.1-mediated CD1a CAR-reconstituted T-cells, illustrating the evident reduction of CD1a^{+} MOLT-4 target cells (*dark blue*).

SI3. Supplementary methods.

SI3.1 Construct sequences.

Construct IMPDH.1^{N}-CD1a scFv:

SIGNAL PEPTIDE; CD1a scFv; INT^{N}; LINKER; HIS$_6$

METDTLLLWVLLLWVPGSTGQVQLQQSGAEVKKPGASVKVSCKASGYAFSTYTMH
WVRQAPGQGLEWMGYINPNSASTSYAQKFQGRVTMTADKSTNTAYMELSSLRSEDT
AVYYCARGFYTMDYWGQGTLVTVSSGGGGSGGGGSGGGGSGGGGSRDIQMTQSPS
SLSASVGDRVTITCQASQDINKYIAWYQFKPGKGPRLLIHYTSTLEPAIPSRFSGSGSGR
EYTFTISSLQPEDIATYYCLHYDNLPWTFGGGTKVEIKRAGGGCFVPGTLVNTENGLK
KIEEIKVGDKVFSHTGKLQEVVDTLIFDRDEEIISINGIDCTKNHEFYVIDKENANRVN
EDNIHLFARWVHAEELDMKKHLLIELEGGGGSGGGGSHHHHHH

Underlined sequence submitted to ALPHAFOLD (**Figure 1**)

Reference vector:

Construct IMPDH.1^{C}-*t*-CAR:

SIGNAL PEPTIDE; HIS$_6$; LINKER; INT^{C}; CD8 HINGE/TMD; 4-1BB; CD3Z; T2A; EGFP

MALPVTGLLLSLGLLLHAARPTGHHHHHHGGGGSGGGGSMKFKLKEITSIETKHYK
GKVHDLTVNQDHSYNVRGTVVHNSICTTTPAPRPPTPAPTIASQPLSLRPEACRPAAG
GAVHTRGLDFACDIYIWAPLAGTCGVLLLSLVITLYCKRGRKKLLYIFKQPFMRPVQT
TQEEDGCSCRFPEEEEGGCELRVKFSRSADAPAYQQGQNQLYNELNLGRREEYDVLD
KRRGRDPEMGGKPQRRKNPQEGLYNELQKDKMAEAYSEIGMKGERRRGKGHDGLY
QGLSTATKDTYDALHMQALPPREGRGSLLTCGDVEENPGPMVSKGEELFTGVVPILV
ELDGDVNGHKFSVSGEGEGDATYGKLTLKFICTTGKLPVPWPTLVTTLTYGVQCFSR
YPDHMKQHDFFKSAMPEGYVQERTIFFKDDGNYKTRAEVKFEGDTLVNRIELKGIDF

KEDGNILGHKLEYNYNSHNVYIMADKQKNGIKVNFKIRHNIEDGSVQLADHYQQNT
PIGDGPVLLPDNHYLSTQSALSKDPNEKRDHMVLLEFVTAAGITLGMDELYK

Underlined sequence submitted to ALPHAFOLD (**Figure 1**)

Reference vector: pCCL

Construct NRDJ.1^{C}-*t*-CAR:

SIGNAL PEPTIDE; HIS_6; LINKER; INT^C; CD8 HINGE/TMD; 4-1BB; CD3Z; T2A; EGFP

MALPVTGLLLSLGLLLHAARPTGHHHHHHGGGGSGGGGSMEAKTYIGKLKSRKIVS
NEDTYDIQTSTHNFFANDILVHNSEITTTPAPRPPTPAPTIASQPLSLRPEACRPAAGGA
VHTRGLDFACDIYIWAPLAGTCGVLLLSLVITLYCKRGRKKLLYIFKQPFMRPVQTTQ
EEDGCSCRFPEEEEGGCELRVKFSRSADAPAYQQGQNQLYNELNLGRREEYDVLDKR
RGRDPEMGGKPQRRKNPQEGLYNELQKDKMAEAYSEIGMKGERRRGKGHDGLYQG
LSTATKDTYDALHMQALPPREGRGSLLTCGDVEENPGPMVSKGEELFTGVVPILVEL
DGDVNGHKFSVSGEGEGDATYGKLTLKFICTTGKLPVPWPTLVTTLTYGVQCFSRYP
DHMKQHDFFKSAMPEGYVQERTIFFKDDGNYKTRAEVKFEGDTLVNRIELKGIDFKE
DGNILGHKLEYNYNSHNVYIMADKQKNGIKVNFKIRHNIEDGSVQLADHYQQNTPI
GDGPVLLPDNHYLSTQSALSKDPNEKRDHMVLLEFVTAAGITLGMDELYK

Reference vector: pCCL

SI3.2. AlphaFold 3 structural modelling and interface-consistency analysis

Model confidence was assessed using metrics provided directly by AF3, including the model ranking score, predicted template modelling score (pTM), interface predicted template modelling score (ipTM), chain-pair ipTM, minimum chain-pair predicted aligned error (PAE), and per-chain pTM. Complete PAE matrices were obtained from the corresponding AF3 full_data output files.

To assess the reproducibility of the predicted protein–protein interface independently of the global AF3 confidence scores, all five predicted models were subjected to cross-model residue-contact analysis. Inter-chain contacts were defined as pairs of residues containing at least one non-hydrogen atom from each chain separated by ≤5.0 Å. Residues participating in at least one such contact were classified as interface residues. For each prediction, sets corresponding to
the interface residues of each interacting chain and to the individual inter-chain residue-pair contacts were generated.

Conservation of the predicted interface across models was quantified using the Jaccard similarity coefficient, defined as $J(A,B) = |A \cap B|/|A \cup B|$, where A and B represent the interface sets obtained from two different models. Jaccard coefficients were calculated

separately for the interface residues of each chain and for the exact inter-chain residue-pair contact maps. All 10 unique pairwise comparisons among the five AF3 models were evaluated, and interface conservation was summarized as the mean and range of the resulting Jaccard coefficients. Contact-map analysis represented the more stringent comparison, as a contact was considered conserved only when the same residue in one chain was predicted to interact with the same residue in the opposing chain in both models.

Confidence in the relative positioning of residues forming the predicted interface was further evaluated using the AF3 PAE matrices. For each residue pair satisfying the ≤5.0 Å structural contact criterion, PAE was evaluated in both directions because PAE is directional. A bidirectional contact-specific PAE was therefore calculated as $[PAE(i,j) + PAE(j,i)]/2$, and the mean contact-pair PAE was calculated across all predicted inter-chain contacts for each model. Lower contact-specific PAE values were interpreted as greater confidence in the relative positioning of residues comprising the predicted interface.

Native AF3 confidence metrics and the derived cross-model interface metrics were considered complementary. The latter, including interface-residue Jaccard coefficients, contact-map Jaccard coefficients, and contact-specific PAE summaries, represent downstream structural analyses and are not native AlphaFold confidence metrics. Predicted interactions were therefore interpreted by considering both AF3 confidence scores and the reproducibility of the interface across the five independently generated structural predictions.

SI3.3. Effects of the protein trans-splicing (PTS) reaction conditions on T-cell viability and activation.

Peripheral blood mononuclear cells (PBMCs) from three healthy donors were transduced with the IMPDH.1^{C}-*t*-CAR construct or left untransduced. To simulate PTS conditions, bovine serum albumin (BSA, 30 μM), taken as an irrelevant protein, was reduced with 5 mM DTT for 1 h at 37 °C and subsequently diluted in complete RPMI medium to a final concentration of 2 μM. Untransduced PBMCs or IMPDH.1^{C}-*t*-CAR T-cells (1×10^5 cells) were incubated with reduced BSA for 3 h (PTS-mimicking conditions). As a positive control for apoptosis and cell death, cells were directly exposed to 2.5 mM DTT for 3 h. Following treatment, cells were washed with PBS and cultured in fresh complete RPMI medium supplemented with hIL-7 and hIL-15 (10 ng/mL each). One replicate was analyzed immediately (3 h) by flow cytometry using Annexin V and 7-AAD, whereas a second replicate was cultured for an additional 24 h

before staining with Annexin V and 7-AAD together with CD25 and CD69 to evaluate cell viability and activation status (Gating strategy **Figure S7**).

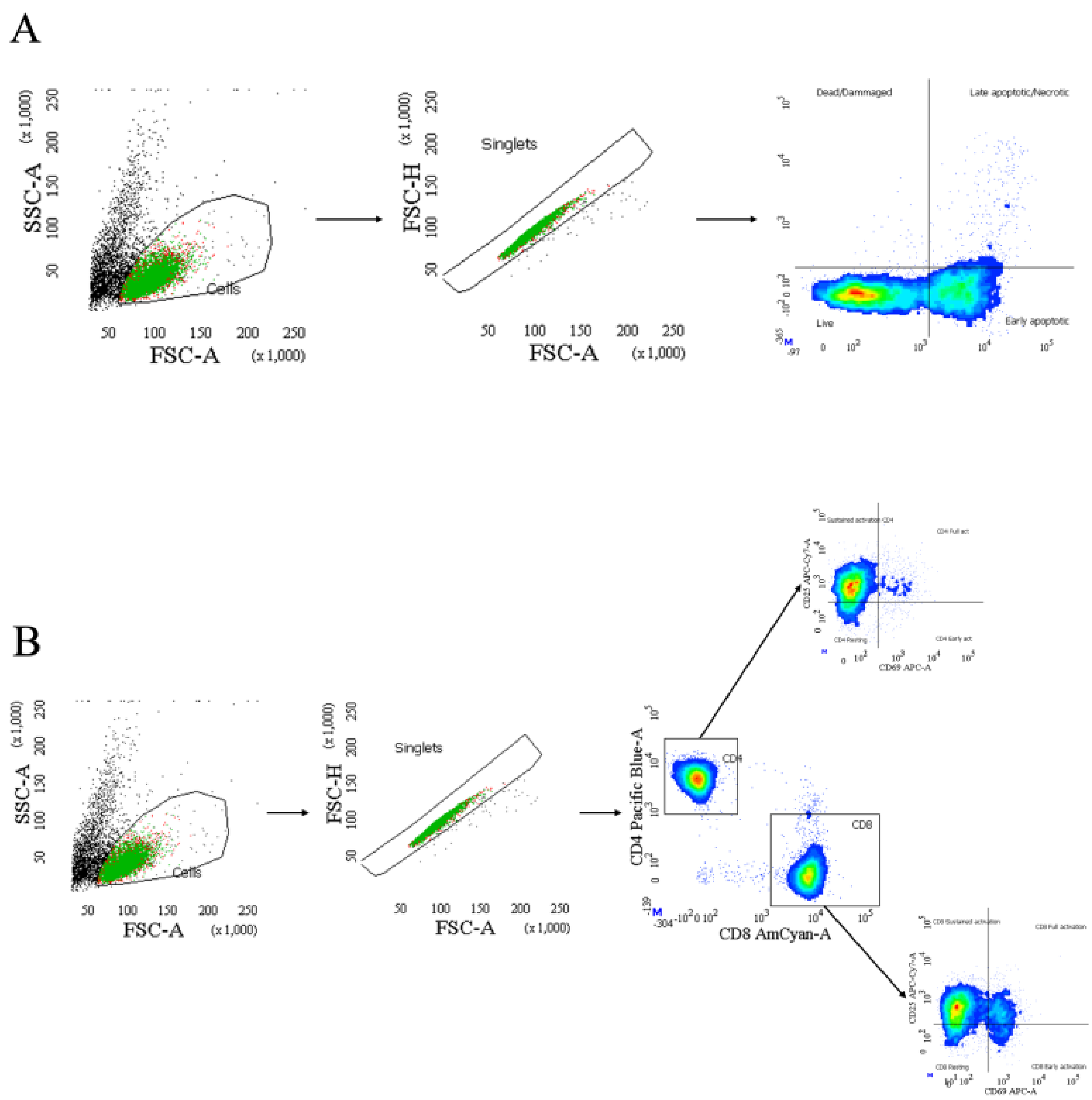

Figure S7. Flow cytometry gating strategy for the analysis of apoptosis and T-cell activation following DTT treatment. (A) Gating strategy used for apoptosis analysis. Cells were first identified based on forward scatter (FSC-A) and side scatter (SSC-A) characteristics, followed by singlet discrimination (FSC-H versus FSC-A). Live, early apoptotic, late apoptotic/necrotic, and dead/damaged cell populations were subsequently identified by Annexin V and 7 Amino Actinomycin D (7-AAD) staining. (B) Gating strategy used for T-cell activation analysis. Cells were initially gated based on FSC-A and SSC-A, followed by singlet discrimination (FSC-H versus FSC-A). $CD4^+$ and $CD8^+$ T-cell populations were then identified, and the expression of the activation markers CD25 and CD69 was assessed within each subset to distinguish resting ($CD25^-CD69^-$), early activated ($CD25^-CD69^+$), fully activated ($CD25^+CD69^+$), and sustained/late activated ($CD25^+CD69^-$) T-cells. Representative gating plots are shown.

SI3.4. Evaluation of basal functionality of IMPDH.1^{C}-*t*-CAR T-cells upon MOLT4 challenge.

T-cells isolated from three healthy donors were transduced to generate IMPDH.1^{C}-*t*-CAR T-cells and analyzed between days 7 and 11 post-transduction. CAR T-cells were cultured alone or co-cultured with CD1a^{+} MOLT4 cells at a 1:1 ratio for 24 or 72 h. Untransduced T-cells stimulated with PMA/ionomycin served as a positive activation control. At each point, cells were analyzed by flow cytometry for activation (CD25, CD69), memory differentiation (CCR7, CD45RA), and exhaustion status (TIM-3, LAG-3, PD-1, and TIGIT) markers on CD4^{+} and CD8^{+} T-cell populations. (Gating strategy **Figure S8**).

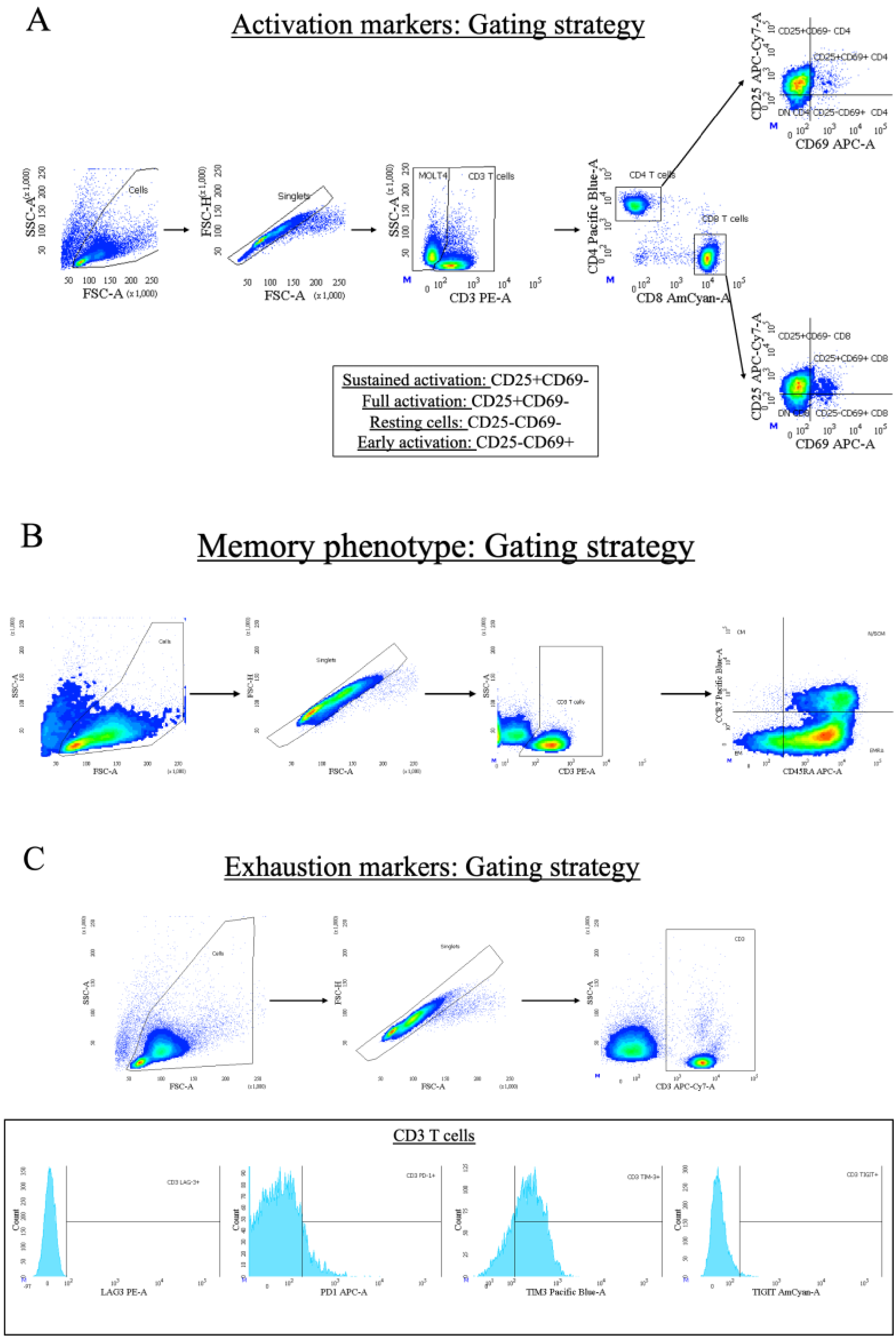
A
Activation markers: Gating strategy
Cells
Singlets
MOLT4
CD3 T cells
CD4 T cells
CD8 T cells
SSC-A
FSC-A
FSC-H
CD3 PE-A
CD4 Pacific Blue-A
CD8 AmCyan-A
CD25 APC-Cy7-A
CD69 APC-A
CD25+CD69- CD4
CD25+CD69+ CD4
DN CD4
CD25-CD69+ CD4
CD25+CD69- CD8
CD25+CD69+ CD8
DN CD8
CD25-CD69+ CD8
Sustained activation: CD25+CD69-
Full activation: CD25+CD69-
Resting cells: CD25-CD69-
Early activation: CD25-CD69+
B
Memory phenotype: Gating strategy
CD3 PE-A
CCR7 Pacific Blue-A
CD45RA APC-A
C
Exhaustion markers: Gating strategy
CD3 APC-Cy7-A
CD3 T cells
Count
LAG3 PE-A
PD1 APC-A
TIM3 Pacific Blue-A
TIGIT AmCyan-A

Figure S8. Flow cytometry gating strategies for the analysis of T-cell activation, memory differentiation, and exhaustion following co-culture with MOLT-4 target cells. (A) Gating strategy for T-cell activation analysis. Cells were initially gated based on forward scatter (FSC-A) and side scatter (SSC-A), followed by singlet discrimination (FSC-H versus FSC-A). $CD3^+$ T-cells were identified to exclude MOLT-4 target cells and subsequently separated into $CD4^+$ and $CD8^+$ T-cell subsets. Within each subset, activation status was determined by the expression of CD25 and CD69, defining resting ($CD25^-CD69^-$), early activated ($CD25^-CD69^+$), fully activated ($CD25^+CD69^+$), and sustained/late activated ($CD25^+CD69^-$) T-cells. (B) Gating strategy for memory phenotype analysis. Following gating on cells, singlets, and $CD3^+$ T-cells, T-cell differentiation subsets were identified based on CD45RA and CCR7 expression as naïve/stemcentral memory (N/SCM; $CD45RA^+CCR7^+$), central memory (CM; $CD45RA^-CCR7^+$), effector memory (EM; $CD45RA^-CCR7^-$), and terminally differentiated effector memory (EMRA; $CD45RA^+CCR7^-$) T-cells. (C) Gating strategy for exhaustion marker analysis. Cells were gated on FSC-A/SSC-A, singlets, and $CD3^+$ T-cells before assessing the surface expression of the inhibitory receptors LAG-3, PD-1, TIM-3, and TIGIT. Representative histograms illustrating the gating strategy for each exhaustion marker are shown. Representative flow cytometry plots are shown for all gating strategies.
